# Chemical synthesis of *Torenia* plant pollen tube attractant proteins by KAHA ligation†

**DOI:** 10.1039/d2cb00039c

**Published:** 2022-03-18

**Authors:** Nandarapu Kumarswamyreddy, Damodara N. Reddy, D. Miklos Robkis, Nao Kamiya, Ryoko Tsukamoto, Masahiro M. Kanaoka, Tetsuya Higashiyama, Shunsuke Oishi, Jeffrey W. Bode

**Affiliations:** Institute of Transformative Bio-Molecules (WPI–ITbM), Nagoya University Chikusa Nagoya 464-8601 Japan bode@itbm.nagoya-u.ac.jp oishi@itbm.nagoya-u.ac.jp; Department of Chemistry, Indian Institute of Technology Tirupati Tirupati Andhra Pradesh 517506 India; Medicinal & Process Chemistry Division, CSIR-Central Drug Research Institute Lucknow 226031 India; Division of Biological Science, Graduate School of Science, Nagoya University Nagoya 464-0602 Japan; Department of Biological Sciences, Graduate School of Science, University of Tokyo Tokyo 113-0033 Japan; Laboratorium für Organische Chemie, Department of Chemistry and Applied Biosciences, ETH Zürich Zürich 8093 Switzerland bode@org.chem.ethz.ch

## Abstract

The synthesis of secreted cysteine-rich proteins (CRPs) is a long-standing challenge due to protein aggregation and premature formation of inter- and intramolecular disulfide bonds. Chemical synthesis provides reduced CRPs with a higher purity, which is advantageous for folding and isolation. Herein, we report the chemical synthesis of pollen tube attractant CRPs *Torenia fournieri* LURE (TfLURE) and *Torenia concolor* LURE (TcLURE) and their chimeric analogues *via* α-ketoacid-hydroxylamine (KAHA) ligation. The bioactivity of chemically synthesized TfLURE protein was shown to be comparable to *E. coli* expressed recombinant protein through *in vitro* assay. The convergent protein synthesis approach is beneficial for preparing these small protein variants efficiently.

## Abstract 2

A convergent chemical synthesis was established for Torenia plant pollen tube attractant proteins, LUREs and their chimeric analogues by KAHA ligation. The synthetic TfLURE showed comparable bioactivity with *E.coli* expressed recombinant protein.

## Graphical abstract

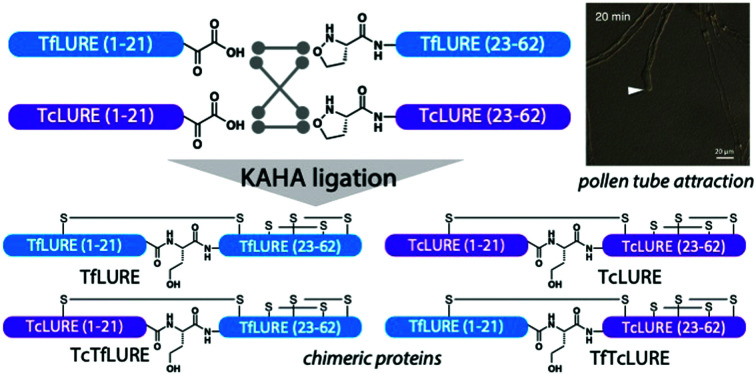

## Introduction

Cysteine-rich proteins (CRPs) are a major class of signaling molecules^1^ that are found across all kingdoms of life including prokaryotes,^2–7^ fungi,^8–13^ plants^14–17^ and animals.^18–22^ Secretory CRPs mediate intercellular signal transduction, which controls cell growth, proliferation, metabolism and many other biological processes.^23–26^ In plants, one important role of CRPs is signal exchange during sexual reproduction.^27–31^ For instance, ovules secrete pollen tube attracting LURE CRPs, which act as a chemoattractant and guide pollen tubes specifically to the ovules.^32–40^ The sequence of LURE proteins is species-specific, and plays a key role in the reproductive barrier between plant species.^41–43^ For example, LURE proteins from *Torenia fournieri* (TfLURE) and *Torenia concolor* (TcLURE) differ in eight amino acid residues in their primary sequences.^43–47^ These differences in the amino acid sequence contribute species-preferential molecular recognition between LURE proteins and the receptors on the pollen tube surface. In other words, the structural difference in LURE proteins is one of the keys for species-specific male–female interactions in plant reproduction.

These small proteins, like all CRPs, contain multiple disulfide bonds that contribute to protein stability and are essential for their biological activities.^48–51^ In the case of TfLURE and TcLURE natural proteins, the connectivity of cysteine residues *via* disulfide bonds has not yet been identified because of difficulties of isolating enough natural proteins from plant pistils. Recombinant expression of CRPs is challenging due to difficulties with the aggregation, precipitation, and identification of the correctly formed disulfide topology of active or natural proteins in the oxidative folding step.^52,53^ These challenges have slowed progress in the investigation of molecular mechanisms of pollen tube attraction due poor access to LURE CRPs and the construction of associated probes. TfLURE and TcLURE can be expressed in *E. coli* and the activity has been demonstrated through *in vitro* pollen tube attraction assays.^43–46^ However, the isomeric purity after *in vitro* oxidative refolding has not been analyzed and the proteins retained a His-tag, which was used for purification. Structurally defined, untagged LURE proteins would benefit from a reliable chemical synthesis that could support quantitative analysis, structure–activity relationship (SAR) studies and site-specific chemical modifications for bioimaging.^54^ Using chemical synthesis, significant quantities of the linear CRPs can be produced, purified and folded under carefully controlled oxidative protein folding conditions. Herein, we document an efficient chemical synthesis of *Torenia* LURE proteins (TfLURE and TcLURE) and their analogues through α-ketoacid-hydroxylamine (KAHA) ligation.

## Results and discussion

### Design

**Fig. 1:**
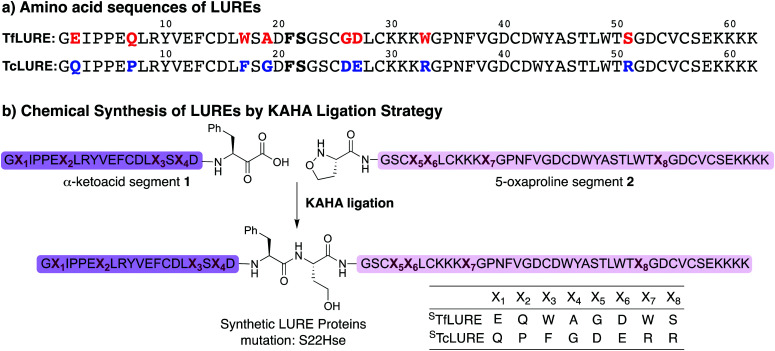
(a) Amino acid sequence of LURE proteins (TfLURE and TcLURE). (b) Proposed synthetic strategy for LUREs by KAHA ligation.

TfLURE and TcLURE contain 62 amino acid residues, differing in eight residues (Fig. 1a, X_1_–X_8_). In addition, they both contain six cysteine residues (Cys14, Cys25, Cys29, Cys41, Cys54 and Cys56) forming three disulfide bonds.

Our initial attempt to synthesize TfLURE and TcLURE *via* 9-fluorenylmethoxycarbonyl-solid phase peptide synthesis (Fmoc-SPPS) as single chains was unsuccessful and promoted to us to switch to a two-fragment α-ketoacid-hydroxylamine (KAHA) ligation strategy. KAHA ligation is the chemoselective ligation of an unprotected peptide fragment containing a C-terminal α-ketoacid with another unprotected peptide fragment containing an N-terminal 5-oxaproline.^55^ The acidic reaction conditions of KAHA ligation are often beneficial for solubilizing the peptide segments. This variant of the ligation strategy leads to the introduction of a non-canonical homoserine (Hse) residue at the ligation site after rearrangement.^56^ Hse differs from canonical serine by an additional methylene group.

Based on the amino sequences of the LUREs, we deemed the linkage between Phe21–Ser22 as suitable for KAHA ligation (see Fig. 1b). The preparation of peptides bearing C-terminal phenylalanine α-ketoacids is well established^57,58^ and the ligation site at this particular position introduces only a minimal substitution of Ser to Hse, which is unlikely to have a strong effect on the protein structure, function, and biological activity.^59–62^

### Protein synthesis

In our preliminary studies we prepared the peptide segments with unprotected cysteine residues, but we observed premature formation and scrambling of disulfide bonds during purification. In order to improve the handling of the peptide segments before refolding, we selected the orthogonal acetamidomethyl (Acm) for Cys protection, which benefits from well-established deprotection protocols.^63^

**Scheme 1:**
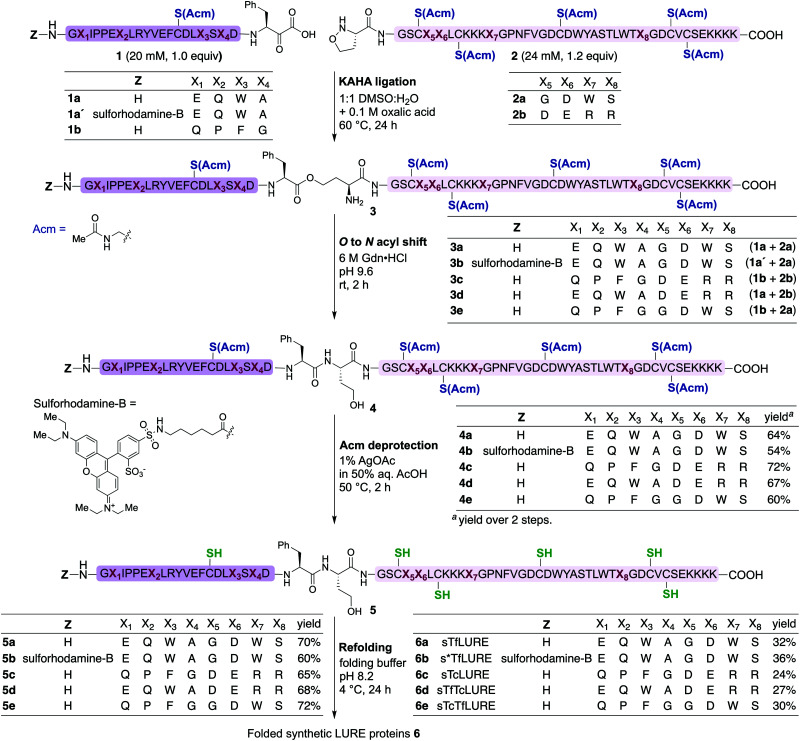
Chemical synthesis of *Torenia* LUREs and analogues *via* the KAHA ligation strategy. KAHA ligation conditions: ligation was performed between the α-ketoacid segment (20 mM, 1.0 equiv.) and the 5-oxaproline segment (24 mM, 1.2 equiv.) in 50% aqueous DMSO with 0.1 M oxalic acid at 60 °C for 24 h. *O*-to-*N* acyl shift conditions: 6 M Gdn·HCl, pH 9.6 at room temperature for 2 h. Acm deprotection conditions: 1% AgOAc in 50% aqueous AcOH at 50 °C for 2 h. Refolding conditions: (i) denature buffer containing 6 M Gdn·HCl with 0.3 M Tris·HCl pH 7.0 at room temperature for 1 h; and (ii) diluted eight-fold with folding buffer containing 5 mM reduced glutathione, 2.5 mM oxidized glutathione, pH 8.2 at 4 °C for 24 h.

We prepared the Cys(Acm)-protected α-ketoacid segments using established Fmoc-SPPS procedures on polystyrene resin preloaded with protected Fmoc-Phe-α-ketoacid.^57,58^ After cleavage of the peptides from the resin with acid, the crude peptides were purified *via* reverse-phase high performance liquid chromatography (RP-HPLC) to obtain pure Cys(Acm)-protected α-ketoacid peptide segments 1a and 1b (Scheme 1) in good yields (16–20% based on the initial resin loading). The Cys(Acm)-protected 5-oxaproline segments were prepared using Fmoc-SPPS on HMPB-ChemMatrix® resin, followed by acidic cleavage and purification *via* RP-HPLC. This provided the desired peptide segments 2a and 2b in good yields (25–30%).

**Fig. 2:**
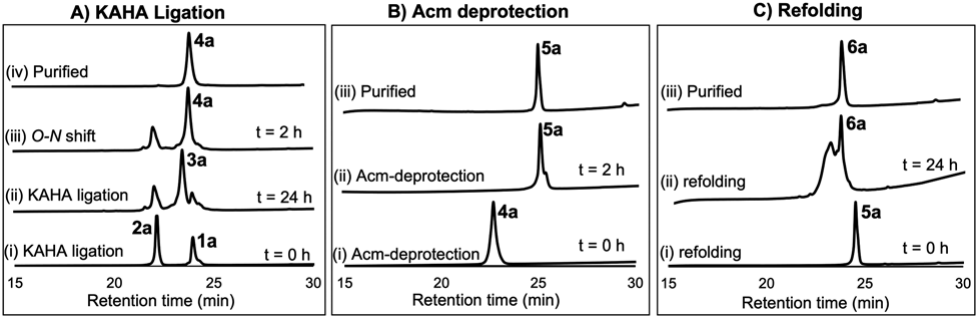
(A) Analytical HPLC traces (*λ* = 220 nm) for KAHA ligation: (i) KAHA ligation at 0 h, (ii) KAHA ligation at 24 h, (iii) *O*-to-*N* acyl shift (depsi/ester peptide to amide rearrangement) at 2 h, and (iv) purified 4a after rearrangement. (B) Analytical HPLC traces (*λ* = 220 nm) for Acm deprotection reaction: (i) Acm deprotection at 0 h, (ii) Acm deprotection at 2 h, and (iii) purified reduced protein 5a. (C) Analytical HPLC traces (*λ* = 220 nm) for refolding: (i) folding at 0 h, (ii) folding at 24 h, and (iii) purified folded protein 6a.

For chemical synthesis of the TfLURE protein through KAHA ligation we coupled 20 mM segment 1a and 24 mM of segment 2a in 50% (v/v) aqueous dimethyl sulfoxide (DMSO) with 0.1 M oxalic acid at 60 °C for 24 h. The KAHA ligation reaction proceeded smoothly with a maximum conversion to give the ligation product 3a. The resulting crude reaction mixture containing depsi-peptide 3a (Scheme 1 and Fig. 2A(ii)) was diluted ten-fold with 6 M guanidine hydrochloride (Gdn·HCl) and the pH was adjusted to 9.6. This induced an *O*-to-*N*-acyl shift to deliver the linear protein 4a. The reaction was monitored using analytical RP-HPLC (Fig. 2A(iii)) and was complete after 2 h. The rearranged protein was purified *via* preparative RP-HPLC to deliver the desired cysteine-protected protein 4a in 64% yield, and the identity was confirmed *via* electrospray ionization high-resolution mass spectrometry (ESI-HRMS) analysis.

The six cysteine Acm protecting groups of protein 4a were removed *via* treatment with 1% AgOAc (w/v) in 50% (v/v) aqueous AcOH at 50 °C. The deprotection reaction proceeded smoothly and the reaction was completed in 2 h. RP-HPLC purification yielded the completely deprotected peptide 5a (Scheme 1 and Fig. 2B(ii)) in reduced form in 70% yield, and the identity was confirmed *via* ESI-HRMS analysis.

Refolding of the denatured protein was performed as previously described.^64^ First, we dissolved the reduced protein 5a at 0.5 mM concentration in denaturation buffer (6 M Gdn·HCl, 0.3 M Tris, pH 7.0) and allowed it to stir at room temperature open to the air.

After one hour, the solution was diluted eight-fold with the folding buffer (5 mM reduced glutathione, 2.5 mM oxidized glutathione, pH 8.2) and stirred at 4 °C for 24 h. We were pleased to see that the major peak *via* analytical RP-HPLC had shifted and resulted in a new sharp peak, indicating the thermodynamically most stable, disulfide-linked, folded TfLURE protein 6a (Scheme 1 and Fig. 2C(ii)). The crude mixture was purified using preparative RP-HPLC and lyophilized to afford pure folded TfLURE protein 6a in 32% yield. The identity of the folded protein was confirmed *via* ESI-HRMS analysis (see Sections 3.4 and 3.5, ESI†). The ESI-HRMS data clearly indicated that the reduced peptide 5a lost a mass equivalent to six protons. This confirms the formation of three disulfide bridges in the folded TfLURE protein 6a.

### Synthesis of rhodamine-labeled TfLURE

Fluorescent labeling is a powerful strategy to study the localization and dynamics of proteins involved in pollen tube guidance.^65,66^ Therefore, we selected sulforhodamine B^67,68^ as a fluorescent dye to attach selectively to the N-terminus of the TfLURE protein sequence. We coupled the sulforhodamine B dye onto the N-terminus of the Cys(Acm)–α-ketoacid segment while it was still on the resin, which was synthesized in an identical manner as 1a. After acidic cleavage of the peptide from the resin, purification *via* RP-HPLC provided the desired sulforhodamine B-labeled peptide segment 1a′ in 12% of yield (see Section S4.1, ESI†).

**Fig. 3:**
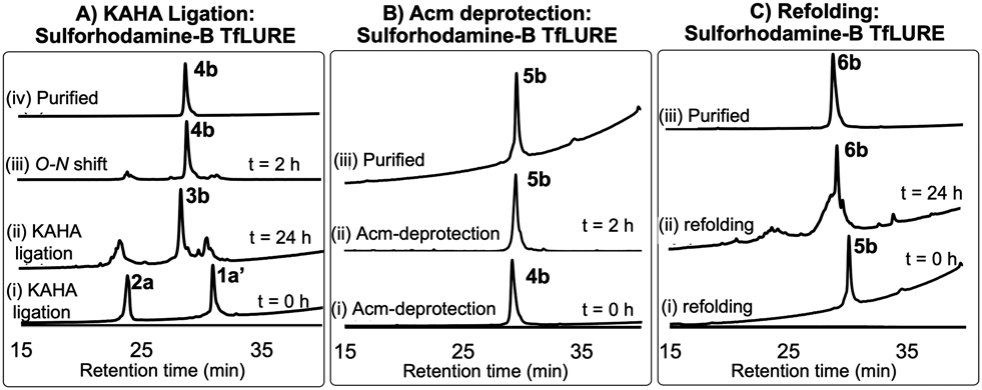
(A) Analytical HPLC traces (λ = 220 nm) for KAHA ligation: (i) KAHA ligation at 0 h, (ii) KAHA ligation at 24 h, (iii) *O*-to-*N* acyl shift (depsi/ester peptide to amide rearrangement) at 2 h, and (iv) purified 4b after rearrangement. (B) Acm deprotection reaction HPLC profiles: (i) Acm deprotection at 0 h, (ii) Acm deprotection at 2 h, and (iii) purified reduced protein 5b. (c) Folding HPLC profiles: (i) folding at 0 h, (ii) folding at 24 h, and (iii) purified folded protein 6b.

Under the optimized KAHA ligation conditions, we performed the ligation reaction between 20 mM of segment 1a′ and 24 mM of segment 2a in 1 : 1 DMSO/water with 0.1 M oxalic acid at 60 °C. The ligation reaction proceeded smoothly within 24 h to yield depsi-peptide 3b (Scheme 1 and Fig. 3A(ii)). The *O*-to-*N*-acyl shift was initiated by diluting ten-fold with 6 M Gdn·HCl, and adjusting the solution to pH 9.6. After 2 h, the reaction mixture was purified using preparative RP-HPLC, which furnished the desired protein 4b in 54% yield (Scheme 1 and Fig. 3A(iii)). Upon Acm deprotection of 4b using 1% AgOAc (w/v) in 50% (v/v) aqueous AcOH for 2 h at 50 °C, we obtained completely deprotected reduced peptide 5b in 60% yield (Scheme 1 and Fig. 3B(ii)).

The reduced peptide 5b was denatured using 6 M Gdn·HCl with 0.3 M Tris buffer pH 7.0 stirred at room temperature for 1 h open to the air, then the protein was folded using our optimized folding conditions by diluting with 8-fold of 5 mM reduced glutathione and 2.5 mM oxidized glutathione set to pH 8.2, then incubation at 4 °C for 24 h. The folded protein was purified *via* RP-HPLC, resulting in the pure folded sulforhodamine B-labeled TfLURE 6b in 36% yield (Scheme 1 and Fig. 3C(iii)), which we further confirmed *via* ESI-MS analysis (see ESI†).

### Bioassay of TfLURE 6a

**Fig. 4:**
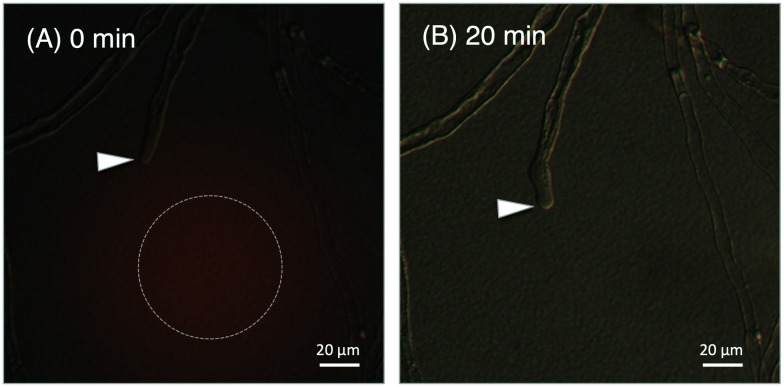
*In vitro* pollen tube attraction assay using synthetic TfLURE 6a, the arrowheads indicate the tips of the pollen tubes. (A) Gelatin beads containing 6a were placed in the dotted circle at 0 min; and (B) diffusion after 20 min.

**Fig. 5:**
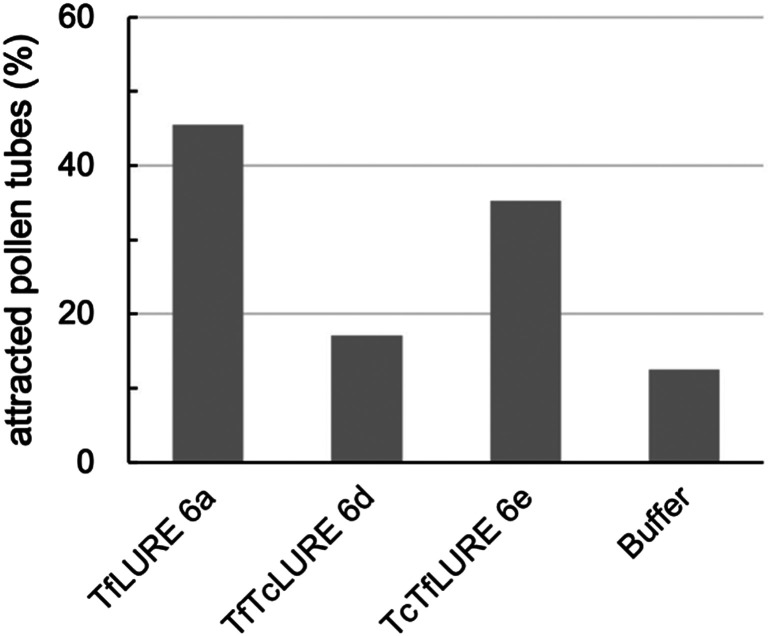
*In vitro* pollen tube attraction assay using synthetic TfLURE 6a (*n* = 11), TfTcLURE 6d (*n* = 34), and TcTfLURE 6e (*n* = 35). 100 nM of proteins were used.

We evaluated the bioactivity of our chemically synthesized TfLURE 6a through *in vitro* pollen tube attraction assays, which have been previously reported.^43,45^ Gelatin beads containing 6a (100 nM) were placed in front of the pollen tube of *Torenia fournieri* (*ca.* 50 μm in distance) and the protein gradually diffused. The synthesized TfLURE 6a attracted 45% (*n* = 11) of pollen tubes (Fig. 4 and 5). Comparable attraction was observed (50%, *n* = 22) with recombinant His-tagged TfLURE proteins. We therefore concluded that the homoserine mutation at the ligation site of synthetic TfLURE 6a did not affect the bioactivity.

### Synthesis of TcLURE and analogues

After the bioassay confirmed that our synthesized protein 6a was active and that the introduction of homoserine did not affect the pollen tube attraction, we sought to synthesize TcLURE. There are eight residues that are different between TfLURE and TcLURE, and these differences are responsible for the species-specific pollen tube attraction. Four of them (X_1_, X_2_, X_3_, X_4_) are embedded in the α-ketoacid segment in the synthetic route and the other residues (X_5_, X_6_, X_7_, X_8_) are in the 5-oxaproline segment (Fig. 1). We also elected to synthesize chimeric proteins (TfTcLURE and TcTfLURE) using our established KAHA ligation strategy. TfTcLURE and TcTfLURE can be prepared *via* exchange of the TfLURE and TcLURE segments 1a, 1b, 2a and 2b shown in Scheme 1.

Under our optimized KAHA ligation and rearrangement conditions, we performed ligation reactions according to segment selection shown in Scheme 1 and synthesized proteins 4c, 4d and 4e in good yields (60–72%). Using our established Acm deprotection conditions, we removed the six Acm groups from 4c, 4d and 4e through treatment with 1% AgOAc in 50% aqueous AcOH for 2 h at 50 °C. The deprotected reduced proteins 5c, 5d, and 5e were isolated in 65–72% yields (Scheme 1). We then performed the folding reaction under our optimized folding conditions for the reduced proteins 5c, 5d, and 5e. The folding proceeded smoothly and produced folded TcLURE 6c, TfTcLURE 6d and TcTfLURE 6e in 24–30% yields after RP-HPLC purification. The final purified folded proteins 6c, 6d and 6e were confirmed *via* ESI-MS analysis (see ESI†).

### Bioassay of protein analogues

We examined TfLURE 6a and the synthetic analogues TfTcLURE 6d and TcTfLURE 6e through an *in vitro* pollen tube attraction assay to elucidate the species-preferentiality in pollen tube attraction. TcTfLURE 6e showed a comparable activity (35%, *n* = 35) to TfLURE 6a (45%, *n* = 11). This suggests that the different residues in the α-ketoacid segment (X_1_, X_2_, X_3_, X_4_) do not strongly contribute to species-preferentiality. On the other hand, TfTcLURE 6d showed a lower attraction activity (17%, *n* = 34). Therefore, residues embedded in the 5-oxaproline segment (*i.e.*, X_5_, X_6_, X_7_, X_8_) appear to be more responsible for the preferentiality in the attraction of *T. fournieri* pollen tubes.

## Conclusions

In conclusion, we developed a versatile synthetic strategy for cysteine-rich pollen tube attractant LURE proteins from *Torenia* through KAHA ligation. The chemically synthesized TfLURE protein 6a showed a comparable attraction of pollen tubes to the recombinant protein. We employed a rapid and efficient convergent synthesis to access the LURE proteins (TfLURE and TcLURE) and their hybrid variants (TfTcLURE and TcTfLURE). Using these proteins, we identified the amino acid residues (Gly26, Asp27, Trp33, and Ser51) responsible for the species-specific pollen tube attraction in *T. fournieri*.

## Author contributions

M. M. K., T. H., S.O. and J. W. B. conceived of the idea. Nandarapu K., D. N. R., and S. O. synthesized the LURE proteins and the analogues. Nandarapu K., D. N. R., and D. M. R. acquired the spectroscopic data of the refolded proteins. Nao K., R. T., and M. M. K. performed the pollen tube attraction assays. M. M. K., T. H., S. O. and J. W. B. designed the experiments, analysed the data, and obtained the funding for this research project. Nandarapu K., S. O., and J. W. B. wrote the manuscript with help from all authors.

## Conflicts of interest

There are no conflicts to declare.

## Supplementary Material

CB-003-D2CB00039C-s001
