# Supplementary material for: Chemical synthesis of Torenia plant pollen tube attractant proteins by KAHA ligation
Source: RSC Chem Biol. 2022 Mar 18;3(6):721–7. doi: 10.1039/d2cb00039c (PMC9175099; doi:10.1039/d2cb00039c)
Supplement: CB-003-D2CB00039C-s001 [file CB-003-D2CB00039C-s001.pdf]

Electronic Supplementary Material (ESI) for RSC Chemical Biology.
This journal is © The Royal Society of Chemistry 2022

Supporting Information

Chemical Synthesis of *Torenia* Plant Pollen Tube Attractant Proteins by KAHA Ligation

Nandarapu Kumarswamyreddy,[a,b] Damodara N. Reddy,[a,c] D. Miklos Robkis[a], Nao Kamiya,[d] Ryoko Tsukamoto,[d] Masahiro M. Kanaoka,[d] Tetsuya Higashiyama,[a,d,e] Shunsuke Oishi,*[a] and Jeffrey W. Bode*[a,f]

a. Institute of Transformative Bio-Molecules (WPI–ITbM), Nagoya University, Chikusa Nagoya 464-8601, Japan. Email: bode@itbm.nagoya-u.ac.jp; oishi@itbm.nagoya-u.ac.jp.

b. Department of Chemistry, Indian Institute of Technology Tirupati, Tirupati, Andhra Pradesh 517506, India.

c. Medicinal & Process Chemistry Division, CSIR-Central Drug Research Institute, Lucknow 226031, India.

d. Division of Biological Science, Graduate School of Science, Nagoya University, Nagoya 464-0602 Japan

e. Department of Biological Sciences, Graduate School of Science, University of Tokyo, Tokyo 113-0033, Japan

f. Laboratorium für Organische Chemie, Department of Chemistry and Applied Biosciences, ETH Zürich, Zürich 8093, Switzerland. Email: bode@org.chem.ethz.ch.

Table of Contents

1. General Methods S2
2. General Experimental Procedures S7
3. Chemical Synthesis of TfLURE by KAHA Ligation S10
4. Chemical Synthesis of Sulforhodamine-B TfLURE by KAHA Ligation S15
5. Chemical Synthesis of TcLURE Protein by KAHA Ligation S20
6. Chemical Synthesis of Analogues of LURE Proteins by KAHA Ligation S26
7. Analysis of LURE Proteins by SDS-Page S32
8. References S33

1. General Methods

1.1. Reagents and solvents and abbreviations

Fmoc-amino acids with suitable side-chain protecting groups, HCTU and HATU were purchased from Merck KGaA (Darmstadt, Germany). HPLC grade CH_3CN from Kanto Chemical Co., Inc. (Tokyo, Japan) and FUJIFILM Wako Pure Chemical Corporation (Osaka, Japan) was used for analytical and preparative HPLC purification. DMF from FUJIFILM Wako Pure Chemical Corporation was directly used without further purification for solid phase peptide synthesis. Fmoc-protected-Phe-α-ketoacid[1] Boc-(*S*)-5-oxaproline[2] were prepared according to reported procedures. Other commercially available reagents and solvents were purchased from Merck KGaA (Darmstadt, Germany), Kanto Chemical Co., Inc. (Tokyo, Japan), FUJIFILM Wako Pure Chemical Corporation (Osaka, Japan), and Tokyo Chemical Industry Co., Ltd. (Tokyo, Japan) and used without further purification.

List of the different building blocks previously reported by our group and other building blocks used in this work:

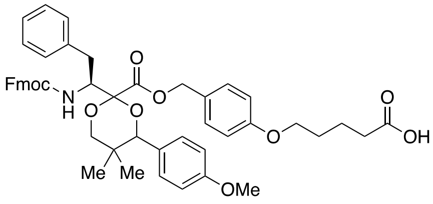

Fmoc-protected-Phe-α-ketoacid

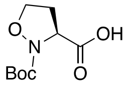

Boc-5-oxaproline (Boc-Opr)

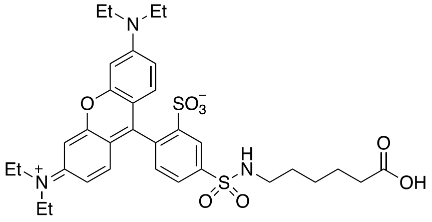

sulforhodamine-B carboxylic acid

List of abbreviations used:

DMSO: dimethylsulfoxide

NMP: *N*-methyl-2-pyrrolidone

DMF: *N*,*N*-dimethylformamide

TFA: trifluoroacetic acid

NMM: *N*-methylmorpholine

HATU: *O*-(7-azabenzotriazol-1-yl)-*N*,*N*,*N'*,*N'*-tetramethyluronium hexafluorophosphate

HCTU: *O*-(1H-6-chlorobenzotriazol-1-yl)-*N*,*N*,*N'*,*N'*-tetramethyluronium hexafluorophosphate

HOBt: hydroxybenzotriazole

DIC: *N*,*N'*-diisopropylcarbodiimide

DODT: 2,2´-(ethylenedioxy)diethanethiol

TIPS: triisopropylsilane

DTT: dithiothreitol

Gdn•HCl: guanidine hydrochloride

Fmoc: 9-fluorenylmethyloxycarbonyl

Pbf: 2,2,4,6,7-pentamethyl-2,3-dihydrobenzofuran-5-yl)sulfonyl

Boc: tert-butoxycarbonyl

Trt: trityl

Acm: acetamidomethyl

MALDI-TOF-MS: Matrix-assisted laser desorption ionization time-of-flight mass spectrometry

SPPS: solid phase peptide synthesis

HMPB: 4-(4-Hydroxymethyl-3-methoxyphenoxy)butyric acid

1.2. Peptide synthesis

a) Automated Solid Phase Peptide Synthesis (SPPS)

Peptides were synthesized on a CS Bio 136X synthesizer using Fmoc SPPS chemistry. The following Fmoc amino acids with side-chain protecting groups were used: Fmoc-Ala-OH, Fmoc-Arg(Pbf)-OH, Fmoc-Asn(Trt)-OH, Fmoc-Asp(OtBu)-OH, Fmoc-Cys(Acm)-OH, Fmoc-Cys(Trt)-OH, Fmoc-Gln(Trt)-OH, Fmoc-Glu(OtBu)-OH, Fmoc-Gly-OH, Fmoc-His(1-Trt)-OH, Fmoc-Ile-OH, Fmoc-Leu-OH, Fmoc-Lys(Boc)-OH, Fmoc-Phe-OH, Fmoc-Pro-OH, Fmoc-Ser(tBu)-OH, Fmoc-Thr(tBu)-OH, Fmoc-Trp(Boc)-OH, Fmoc-Tyr(tBu)-OH, Fmoc-Val-OH. SPPS was performed on aminomethyl polystyrene resin or HMPB-ChemMatrix resin. Manual loading of the first amino acid residue onto the resin and subsequent Fmoc-SPPS followed established standard protocols. A brief summary of the utilized synthesis protocols: Fmoc-deprotections were performed with 20%(v/v) piperidine in DMF (8 min × 2). Couplings were performed with Fmoc-amino acid (4.0 equiv relative to resin substitution), HCTU (3.8 equiv) and NMM (8.0 equiv) in DMF for 60 min. If required, the coupling step was repeated (double coupling) and LiCl washes (0.8 M LiCl in DMF) were performed before Fmoc-deprotection and coupling. After coupling, unreacted free amine was capped by treatment with 20% (v/v) acetic anhydride and 10% (v/v) NMM in DMF for 10 min. Amino acid residues prone to epimerization such as cysteine were coupled using preformed HOBt esters. In a typical procedure, Fmoc-Cys(Acm)-OH (4.0 equiv relative to resin loading) was dissolved in DMF, and HOBt (4.0 equiv) and DIC (4.0 equiv) were added. The mixture was added to the resin and allowed to react for 2 h.

b) Manual coupling of special amino acids

Valuable non-standard monomers (e.g. Boc-5-oxaproline: Boc-Opr) were coupled manually. The monomer (1.5 equiv) was dissolved in a minimal amount of anhydrous DMF (the minimal concentration of the monomer was 0.1 M), HATU (1.5 equiv) and NMM (3.0 equiv) were added. After a brief period of preactivation (2 min), the solution was added to the resin and allowed to react for 2 h. If required, the coupling was repeated with 1.0 equiv of monomer, 1.0 equiv of HATU, and 2.0 equiv of NMM.

1.3. General HPLC analysis and purification

Peptides and proteins were analyzed and purified by reverse phase high performance liquid chromatography (RP-HPLC) on JASCO analytical and preparative instruments equipped with dual pumps, a mixer, an in-line degasser, and a variable wavelength UV detector (simultaneous monitoring of the eluent at 220 nm, 254 nm and 301 nm) or on a Gilson preparative instrument fitted with a 10 mL injection loop. If required, the columns were heated using a column heater or a water bath. The mobile phase for RP-HPLC were Milli-Q water containing 0.1% TFA and HPLC grade CH_3CN containing 0.1% TFA. In the described HPLC analysis and purifications, TFA was always used as solvent modifier.

Analytical RP-HPLC: Analytical HPLC was performed on a Shiseido Capcell Pak C18 MG-II (5 μm, 120 Å pore size, 4.6 mm I.D. × 250 mm), on a Phenomenex Jupiter C18 (5 μm, 300 Å pore size, 4.6 mm I.D. × 250 mm), or on a Phenomenex Jupiter C4 (5 μm, 300 Å pore size, 4.6 mm I.D. × 250 mm) at a flow rate of 1 mL/min.

Preparative RP-HPLC: Preparative HPLC was performed on a Shiseido Capcell pak C18 UG80 (50 mm I.D. × 250 mm), on a Phenomenex Jupiter C18 (5 μm, 300 Å pore size, 30 mm I.D. × 250 mm), on a Phenomenex Jupiter C18 (5 μm, 300 Å pore size, 21.2 mm I.D. × 250 mm), on a Phenomenex Jupiter C4 (5 μm, 300 Å pore size, 30 mm I.D. × 250 mm), or on a Phenomenex Jupiter C4 (5 μm, 300 Å pore size, 21.2 mm I.D. × 250 mm). The following type of method was used: the column was pre-equilibrated at starting solvent composition for typically 10 min. After injection of the sample, the solvent composition was run to the final solvent composition (e.g., 50% CH_3CN). After the gradient run time, the solvent composition was changed to 95% CH_3CN within 1 min and the column was flushed for 5–7 min. Within 1 min, the solvent composition was changed to 10% CH_3CN and the run ended. For the sake of simplicity, only the gradient time, the starting and end composition of the eluent will be stated at the individual experiments, although all experiments included the full cycle as described above.

1.4. Characterization

MALDI-TOF-MS data were obtained on a Bruker Microflex MALDI-TOF spectrometer using 4-hydroxy-α-cyanocinnamic acid as matrix. High-resolution mass spectra were recorded by the

Molecular Structure Center at ITbM, Nagoya University on a Thermo Scientific™ Exactive™ Plus Orbitrap Mass Spectrometer. SDS-PAGE was carried out on 16.5% acrylamide gels using the Mini-PROTEAN electrophoresis system (Bio-Rad Laboratories) on Precision Plus Protein™ Dual Xtra Prestained Protein Standards with Coomassie Brilliant Blue R250 stain.

2. General Experimental Procedures

2.1. General Experimental Procedure for the Synthesis of α-Ketoacid Segment 1

The α-ketoacid segment **1** were synthesized on aminomethyl polystyrene resin preloaded with Fmoc-protected-Phe α-ketoacid (0.30–0.35 mmol/g loading) by automated Fmoc SPPS. To the resin placed in a glass vial, a mixture of 95:2.5:2.5 TFA:DODT:H_2O (20 mL/g resin) was added and the suspension was shaken at room temperature. After 2 h, the crude TFA solution was separated from the resin by filtration and the filtrate was concentrated under reduced pressure. The residue was triturated with cooled Et_2O (ca. 15 mL/g resin), centrifuged and the supernatant was removed by decantation. This trituration/washing step was repeated twice. The crude peptide was dried and dissolved in a suitable solvent (aqueous CH_3CN or aqueous CH_3CN/DMF or AcOH with 0.1% TFA) for RP-HPLC purification.

2.2. General Experimental Procedure for the Synthesis of 5-Oxaproline Segment 2:

5-Oxaproline segment **2** was prepared on HMPB-ChemMatrix resin preloaded with Fmoc-Lys(Boc)-OH (0.25–0.30 mmol/g loading). After automated Fmoc SPPS, Boc-Opr (1.5 equiv) was coupled at N-terminus using HATU (1.4 equiv) and NMM (3.0 equiv) in DMF for 4 h at room temperature to complete the SPPS. The resin was washed several times with DMF followed by CH_2Cl_2 and subjected to cleavage using a mixture of 95:2.5:2.5 TFA:TIPS:H_2O (20 mL/g resin) for 2 h at room temperature. The crude TFA solution was separated from the resin by filtration and the filtrate was concentrated under reduced pressure. The residue was triturated with cooled Et_2O (ca. 25 mL/g resin), centrifuged and the supernatant was removed by decantation. This trituration/washing step was repeated twice. The crude peptide was dried and dissolved in aqueous CH_3CN with 0.1% TFA for RP-HPLC purification.

2.3. Optimization of the KAHA Ligation Conditions using 1a and 2a.

Various reaction conditions were examined using **2a** (1.0 equiv, 20 mM) and **1a** (1.2 equiv, 24 mM) in 0.1 M oxalic acid solution (Table 1). The reactions were analyzed by analytical RP-HPLC.

Entry	solvent	temp.	time	Yield of **3a**[a]
1	NMP/H_2O (5:1)	50 °C	7 h	8%
2	NMP/H_2O (5:1)	55 °C	7 h	0%[b]
3	NMP/H_2O (5:1)	60 °C	7 h	0%[b]
4	NMP/H_2O (1:1)	50 °C	72 h	42%
5	NMP/H_2O (1:1)	55 °C	6.5 h	18%
6	NMP/H_2O (1:1)	60 °C	4.5 h	10%
7	DMSO/H_2O (5:1)	50 °C	12 h	27%
8	DMSO/H_2O (5:1)	55 °C	12 h	32%
9	DMSO/H_2O (5:1)	60 °C	10 h	40%
10	DMSO/H_2O (1:1)	50 °C	24 h	37%
11	DMSO/H_2O (1:1)	55 °C	24 h	51%
12	DMSO/H_2O (1:1)	60 °C	24 h	64%

Table S1: Optimization of the KAHA Ligation using 1a and 2a [a] ligation product conversion determined by analytical RP-HPLC. [b]decomposition of α-ketoacid segment **1a** was observed during KAHA ligation.

In the case of NMP/H_2O as solvent, the C-terminal α-ketoacid of **1a** decomposed into carboxylic acid. The formal decarbonylation was observed at 55 °C and was reduced at 60 °C (entries 2 and 3), however we did not observe complete conversion for the ligated *depsi* peptide **3a**, even longer reaction time (entry 4). To improve the yield, we switched to DMSO/water solvent system with changing the ratio (entries 7–12). We found that 50% aqueous DMSO and 60 °C were suitable for the KAHA ligation to produce the desired *depsi* peptide **3a** (entry 12).

2.4. General Experimental Procedure for the KAHA Ligation and Rearrangement:

5-Oxaproline segment **2** (1.0 equiv) and α-ketoacid Segment **1** (1.2 equiv) were weighed into a glass vial and dissolved in a mixture of 1:1 DMSO/H_2O (20 mM concentration of **2**) with 0.1 M oxalic acid. The mixture was heated to 60 °C for 24 h. After 24 h, the crude *depsi*-peptide was subjected to *O*-to-*N* acyl shift (rearrangement) by dilution to 10-fold volume with 6 M Gdn•HCl

solution set to pH 9.6, and the mixture was stirred at room temperature for 2 h. The reaction mixture was purified by preparative RP-HPLC.

2.5. General Experimental Procedure for the Cysteine Acm-deprotection:

The cysteine-Acm protected protein (10 mg) was dissolved in 2.5 mL of 50% (v/v) aq. acetic acid containing 1% AgOAc (w/v) and the mixture was stirred at 50 °C for 2 h. The reaction was quenched with 4.0 mL of 10% DTT (x/v) in 50% aq. acetic acid, and precipitation was separated by centrifugation. The precipitate was repeatedly washed with 50% (v/v) aq. acetic acid solution and the combined supernatant was purified by preparative RP-HPLC.

2.6. General Experimental Procedure for Protein Folding:

The deprotected, reduced linear protein (1 mg) was dissolved in denaturing buffer containing 6 M Gdn•HCl and 0.3 M Tris•HCl buffer, pH 7.0 (0.5 mM peptide concentration) stirred at room temperature to open air. After 1 h, the mixture was diluted with 8-fold volume of folding buffer containing 5 mM of reduced glutathione and 2.5 mM oxidized glutathione and adjusted to pH 8.2. The reaction was incubated in a shaker with slow movement at 4 °C for 24 h. The folding progress was monitored by analytical RP-HPLC. The resulting solution was acidified with aqueous HCl adjust to pH 4–5 and purified by preparative HPLC.

3. Chemical Synthesis of TfLURE by KAHA Ligation

3.1. Synthesis of Cys(Acm) Protected α-Ketoacid Peptide 1a

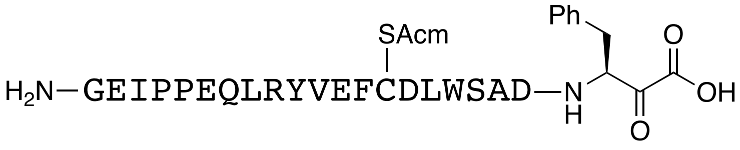

1a

Peptide **1a** was synthesized according to General Procedure 2.1. The crude peptide was purified by preparative RP-HPLC using a Shiseido Capcell pak C18 UG80 (50 mm I.D. × 250 mm) at room temperature with a gradient of 15–65% CH_3CN (with 0.1% TFA) in 20 min, flow rate 40 mL/min and re-purified using a Phenomenex Jupiter C4 (5 μm, 300 Å pore size, 30 mm I.D. × 250 mm) at room temperature with a gradient of 20–60% CH_3CN (with 0.1% TFA) in 20 min, flow rate 20 mL/min. The fractions containing the desired product were pooled and lyophilized to give 83 mg of peptide **1a** (obtained from 1 g of dried resin after SPPS). The purity and identity of **1a** was confirmed using analytical HPLC and ESI-HRMS. m/z calculated for **1a** $C_{120}H_{171}N_{27}O_{37}S$ $[M+2H]^{2+}$: 1307.1019 Da, measured: 1307.0998 Da.

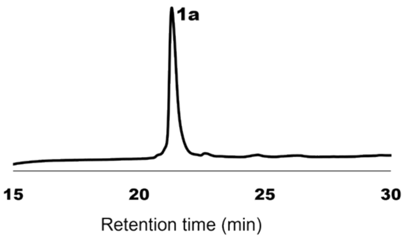

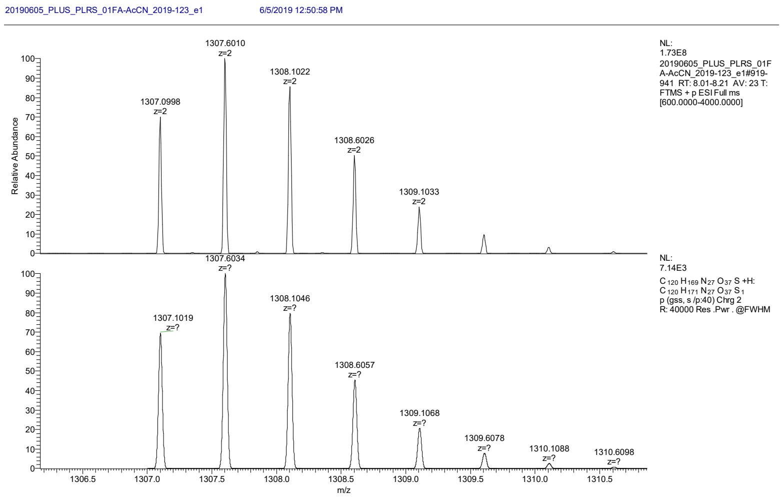

3.2. Synthesis of Cys(Acm) Protected 5-Oxaproline Peptide 2a

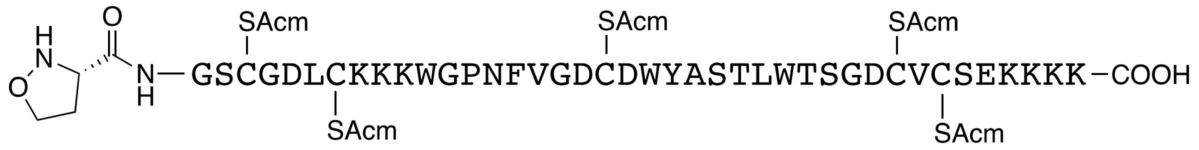

2a

Peptide **2a** was synthesized according to General Procedure 2.2. The crude peptide was purified by preparative RP-HPLC using a Shiseido Capcell pak C18 UG80 (50 mm I.D. × 250 mm) at room temperature with a gradient of 15–55% CH_3CN (with 0.1% TFA) in 20 min, flow rate 40 mL/min and re-purified by a Phenomenex Jupiter C18 (5 µm, 300 Å pore size, 21.2 mm I.D. × 250 mm) at room temperature with a gradient of 15–50% CH_3CN (with 0.1% TFA) in 20 min, flow rate 10 mL/min. The fractions containing the desired product were pooled and lyophilized to give 97 mg of desired 5-oxaproline peptide **2a** (obtained from 1 g of dried resin after SPPS). The purity and identity of **2a** was confirmed using analytical HPLC and ESI-HRMS. m/z calculated for peptide **2a** $C_{212}H_{327}N_{57}O_{66}S_5[M+4H]^{4+}$: 1221.8141 Da; measured 1221.8126 Da.

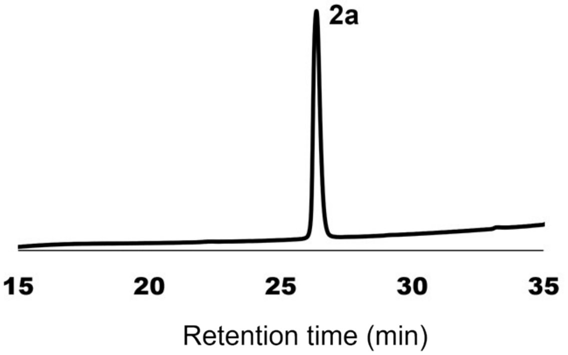

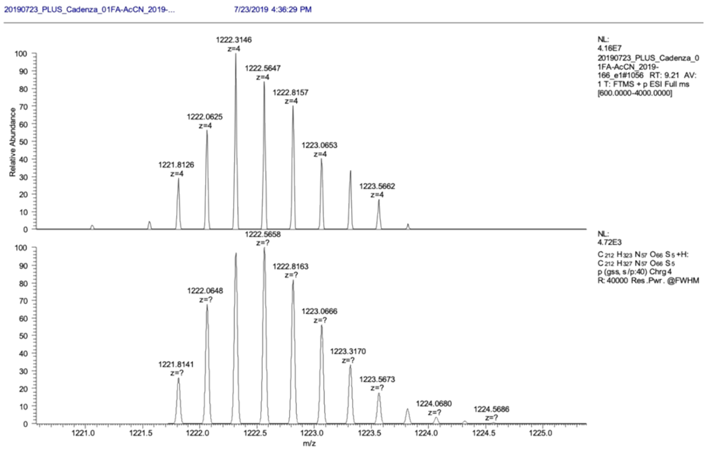

3.3. Synthesis of Cys(Acm) Protected Linear Protein 4a by KAHA ligation

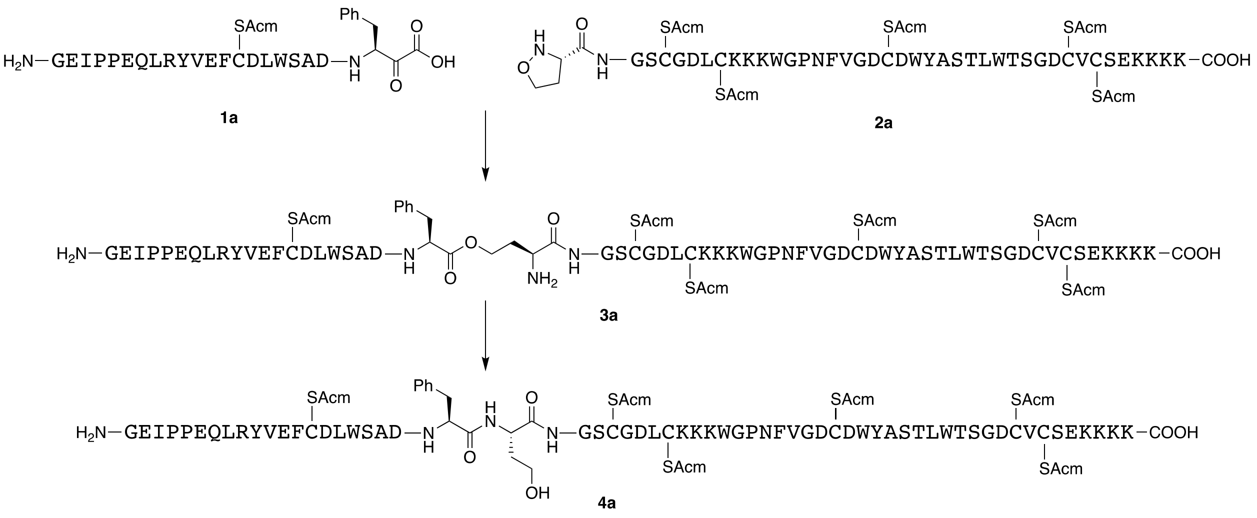

Linear protein **4a** was synthesized according to General Procedure 2.4 using peptide **2a** (30 mg, 6.5 μmol, 1.0 equiv) and peptide **1a** (42 mg, 7.8 μmol, 1.2 equiv).The reaction mixture was purified by preparative RP-HPLC using a Phenomenex Jupiter C18 (5 μm, 300 Å pore size, 30 mm I.D. × 250 mm), heated to 60 °C; with a gradient of 10–60% CH_3CN with 0.1% TFA in 30 min, flow rate 20 mL/min. The fractions containing the desired product were pooled and lyophilized to give pure **4a** (29 mg, 64% yield). The purity and identity of **4a** was confirmed using analytical HPLC and ESI-HRMS. The m/z calculated for **4a** $C_{331}H_{497}N_{84}O_{101}S_6$ $[M+5H]^{5+}$: 1491.4932 Da; measured 1491.4903 Da.

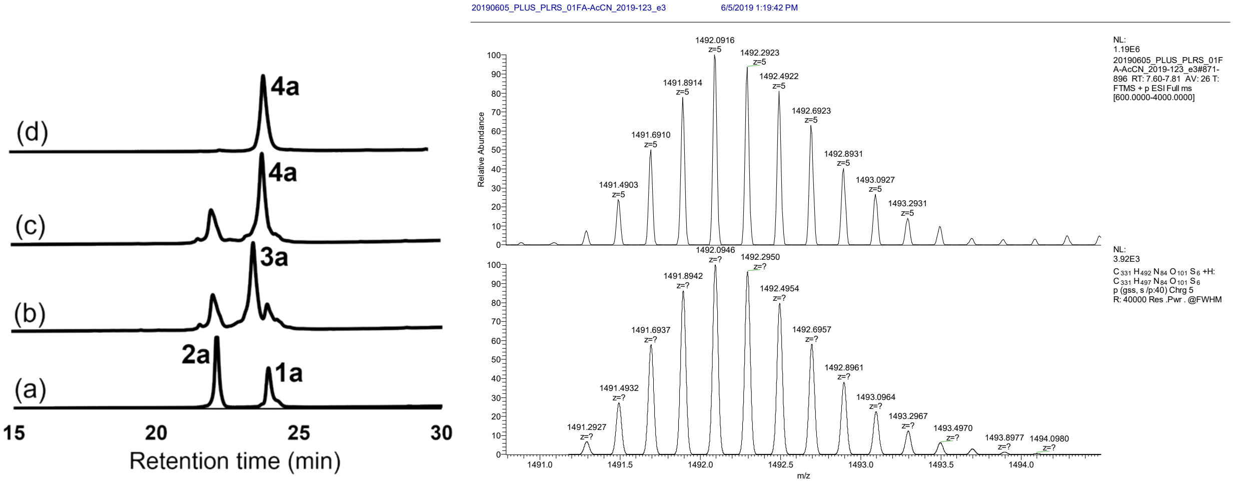

(a) reaction time t = 0 h; (b) reaction time t = 24 h; (c) 2 h after addition of 6M Gdn•HCl solution pH 9.6; (d) purified **4a**.

3.4. Synthesis of Linear Protein 5a

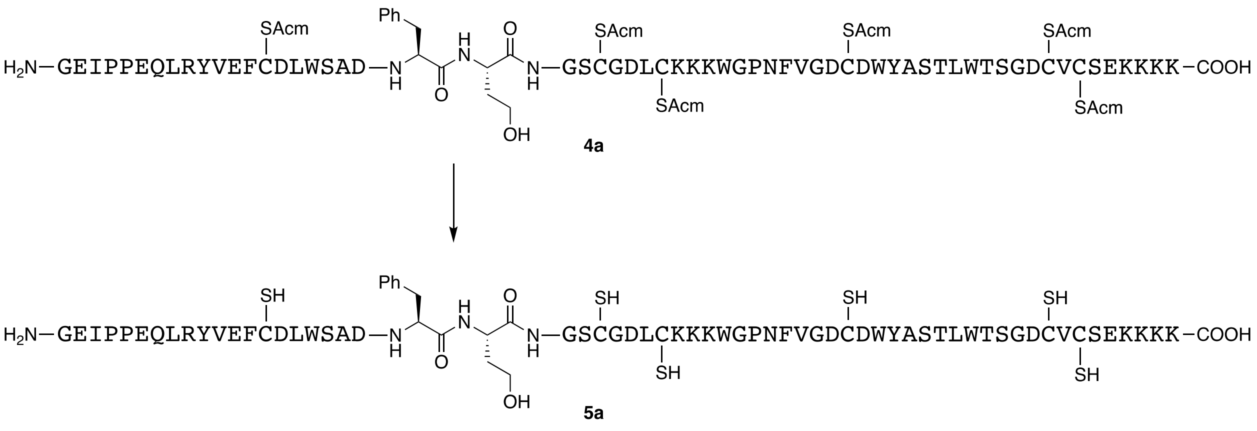

Reduced linear protein **5a** was synthesized according to General Procedure 2.5 with Cys(Acm) protected protein **4a** (10 mg, 14 µmol, 1.0 equiv). The obtained mixture was purified by preparative HPLC using a Phenomenex Jupiter C18 (5 µm, 300 Å pore size, 21.2 mm I.D. × 250 mm), heated to 60 °C, with a gradient of 30–80% CH_3CN with 0.1% TFA in 30 min, flow rate 10 mL/min. The fractions containing the desired product were pooled and lyophilized to give pure **5a** (6.6 mg, 70% yield). The purity and identity of **5a** was confirmed using analytical HPLC and ESI-HRMS. The m/z calculated for **5a** $C_{313}H_{469}N_{78}O_{95}S_6[M+7H]^{7+}$: 1004.7512 Da; measured 1004.7509 Da.

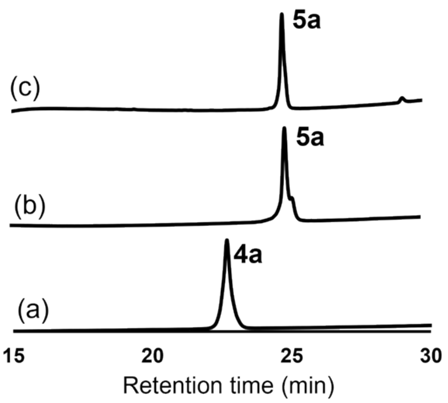

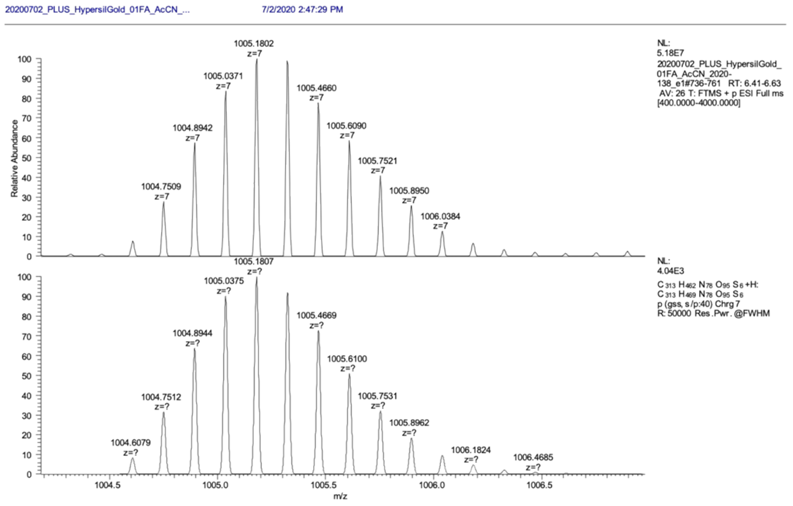

(a) reaction time t = 0 h; (b) reaction time t = 2 h; (c) purified **5a**

3.5. Synthesis of Folded TfLURE Protein 6a

Folded TfLURE protein **6a** was synthesized according to General Procedure 2.6 with 1 mg of reduced linear protein **5a**. The resulting solution was purified by preparative HPLC using a Phenomenex Jupiter C18 (5 µm, 300 Å pore size, 21.2 mm I.D. × 250 mm) at room temperature CH_3CN with 0.1% TFA in 30 min, flow rate 10 mL/min. The fractions containing the desired product were pooled and lyophilized to give pure folded TfLURE **6a** (0.32 mg, 32% yield). The purity and identity of **6a** was confirmed using analytical HPLC and ESI-HRMS. The m/z calculated for folded TfLURE protein **6a** $C_{313}H_{457}N_{78}O_{95}S_6$ $[M+5H]^{5+}$: 1405.0393 Da; measured 1405.0375 Da.

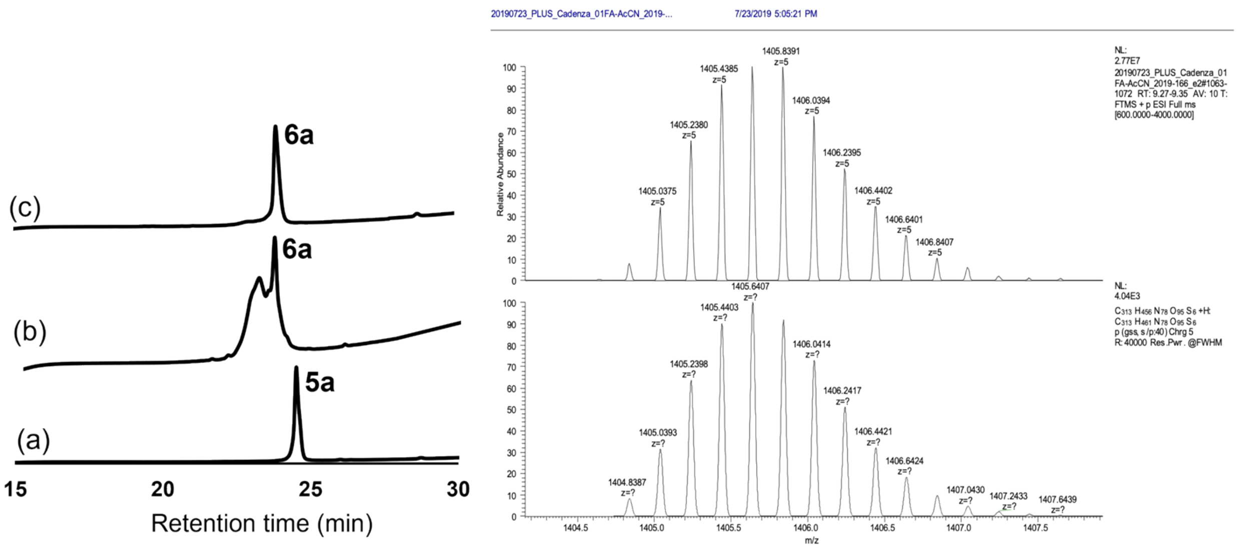

(a) reaction time t = 0 h; (b) reaction time t = 24 h; (c) purified **6a**.

Folded TfLURE protein 6a was dissolved in 25 mM of potassium phosphate buffer (pH = 7.0) and the CD spectrum was measured by a J-1500 spectropolarimeter (JASCO, Tokyo) at room temperature using 0.1 cm cell.

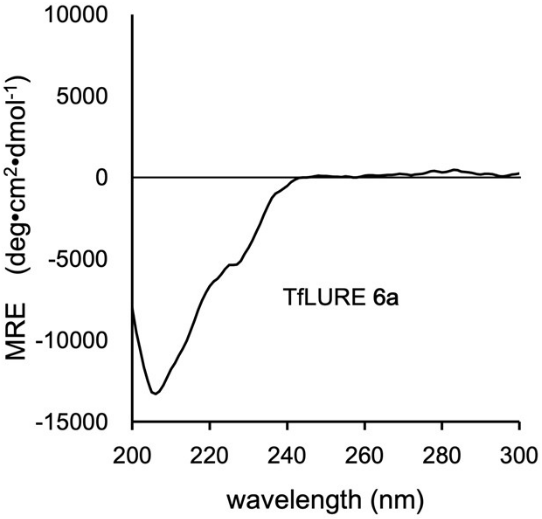

4. Chemical Synthesis of Sulforhodamine-B TfLURE by KAHA Ligation

4.1. Synthesis of Sulforhodamine-B Cys(Acm) Protected α-Ketoacid Peptide 1a′

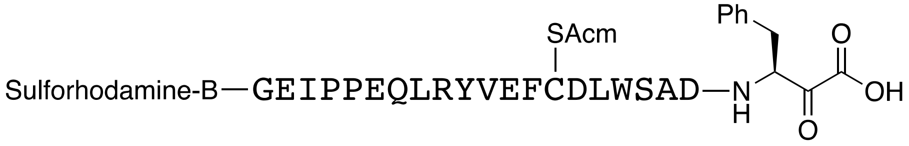

1a′

Sulforhodamine-B =

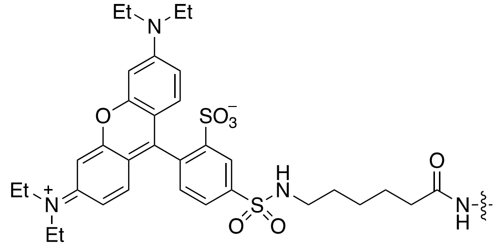

Sulforhodamine B α-ketoacid peptide **1a′** was synthesized according to General Procedure 2.1 on 0.30 mmol scale. After automated Fmoc SPPS, functionalized sulforhodamine-B carboxylic acid (1.1 equiv, 0.33 mmol) in anhydrous DMF was manually coupled with HATU (1.1 equiv, 0.33 mmol), NMM (2.2 equiv, 0.66 mmol) for 2 h at room temperature. The crude peptide was obtained after TFA cleavage described in General Procedure 2.1. The crude peptide was purified by preparative HPLC using a Shiseido Capcell pak C18 UG80 (50 mm I.D. × 250 mm) at room temperature with a gradient of 15–65% CH_3CN in 20 min, flow rate 40 mL/min and re-purified using a Phenomenex Jupiter C4 (5 μm, 300 Å pore size, 30 mm I.D. × 250 mm) at room temperature

with a gradient of 20–60% CH_3CN (with 10% DMF and 0.1% TFA) in 20 min, flow rate 20 mL/min. The fractions containing the desired product were pooled and lyophilized to give 33 mg of sulforhodamine B peptide **1a′** (obtained from 1 g of dried resin after SPPS). The purity and identity of **1a′** was confirmed using analytical HPLC and ESI-HRMS. m/z calculated for **1a′** $C_{153}H_{210}N_{30}O_{44}S_3$ $[M+2H]^{2+}$: 1633.7134 Da, Found: 1633.7108 Da.

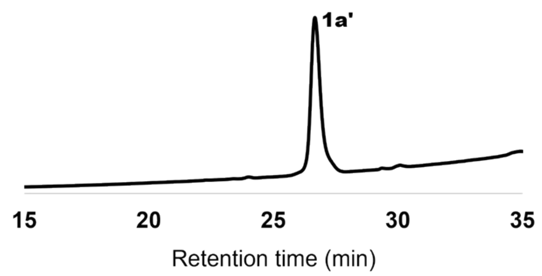

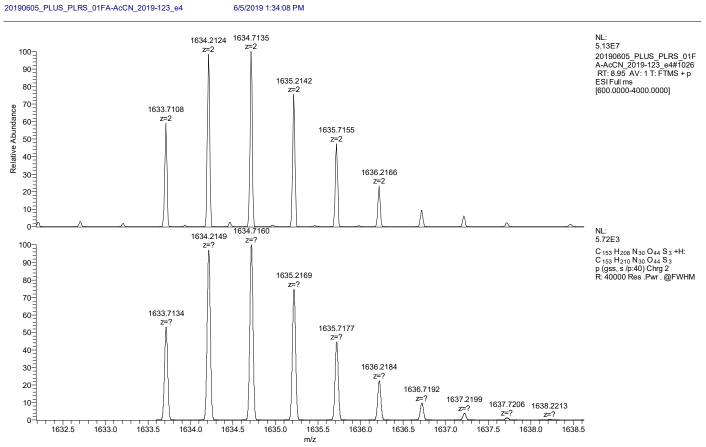

4.2. Synthesis of Sulforhodamine-B Cys(Acm) Protected Protein 4a by KAHA Ligation

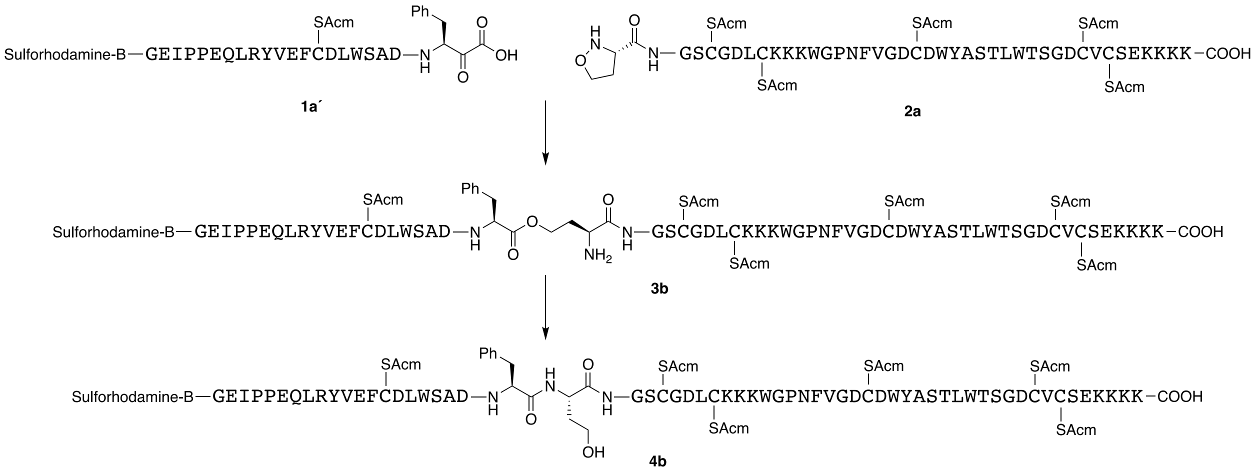

Peptide **4b** was synthesized according to General Procedure 2.4 using peptide **2a** (30 mg, 6.1 µmol, 1.0 equiv) and sulforhodamine-B peptide **1a′** (24 mg, 7.4 µmol, 1.2 equiv). The reaction mixture was purified by preparative RP-HPLC using on a Phenomenex Jupiter C4 (5 µm, 300 Å pore size, 30 mm I.D. × 250 mm) at room temperature with a gradient of 10–60% CH_3CN with 0.1% TFA in 30 min, flow rate 20 mL/min. The fractions containing the desired product were pooled and lyophilized to give pure **4b** (27 mg, 54% yield). The purity and identity of **4b** was confirmed using

analytical HPLC and ESI-HRMS. The m/z calculated for **4b** $C_{364}H_{537}N_{87}O_{108}S_8$ $[M+6H]^{6+}$: 1351.9494 Da; measured 1351.9446 Da.

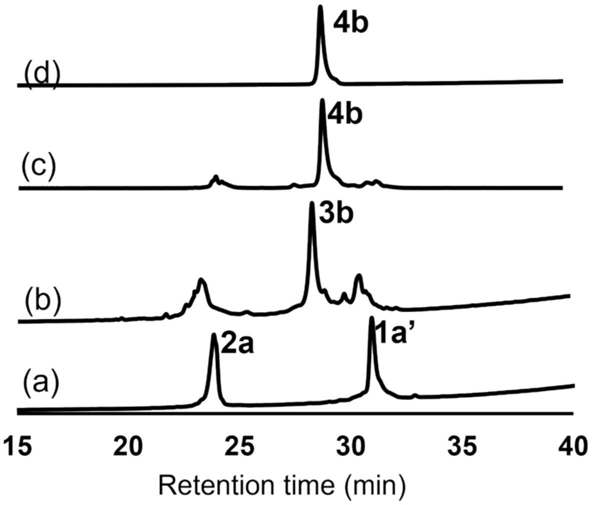

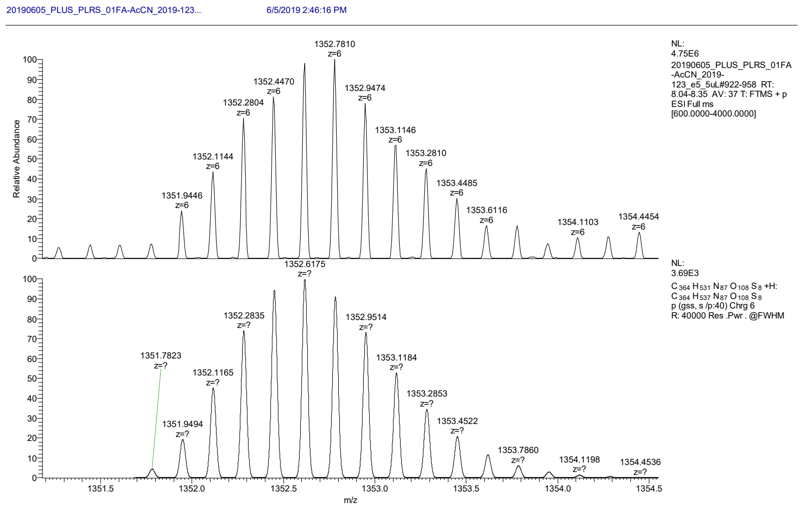

(a) reaction time t = 0 h; (b) reaction time t = 24 h; (c) 2 h after addition of 6M Gdn•HCl solution pH 9.6; (d) purified **4b**.

4.3. Synthesis of Sulforhodamine-B Linear Protein 5b

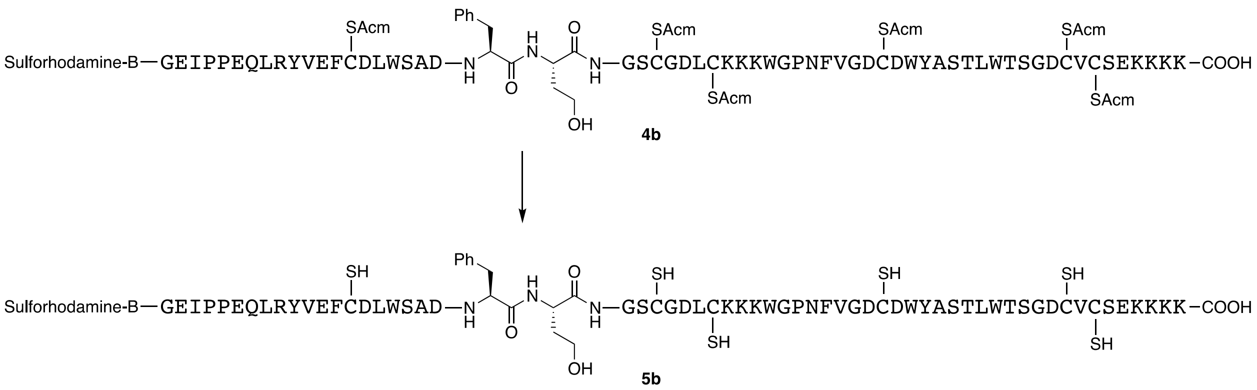

Sulforhodamine-B reduced linear protein **5b** was synthesized according to General Procedure 2.5 with Cys(Acm) protected protein **4b** (10 mg, 13 μmol, 1.0 equiv). The obtained mixture was purified by preparative HPLC using a Phenomenex Jupiter C4 (5 μm, 300 Å pore size, 21.2 mm I.D. × 250 mm), with a gradient of 10–60% CH_3CN with 0.1% TFA in 30 min, flow rate 10 mL/min. The fractions containing the desired product were pooled and lyophilized to give pure reduced protein **5b** (5.7 mg, 60% yield). The purity and identity of **5b** was confirmed using analytical HPLC and ESI-HRMS. The m/z calculated for **5b** $C_{346}H_{507}N_{81}O_{102}S_8$ $[M+6H]^{6+}$: 1280.7451 Da; measured 1280.7416 Da.

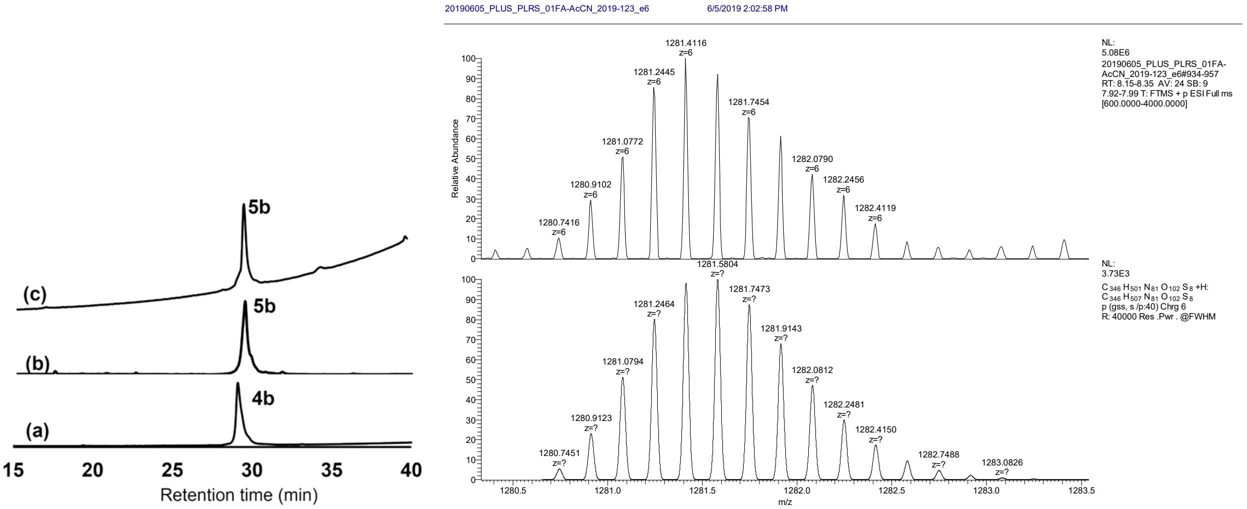

(a) reaction time t = 0 h; (b) reaction time t = 2 h; (c) purified **5b**.

4.4. Synthesis of Folded Sulforhodamine B TfLURE Protein 6b

Folded Sulforhodamine B TfLURE protein **6b** was synthesized according to General Procedure 2.6 with 1 mg of reduced protein **5b**. The resulting solution was purified by preparative HPLC using a Phenomenex Jupiter C4 (5 µm, 300 Å pore size, 21.2 mm I.D. × 250 mm) at room temperature with a gradient of 10–60% CH_3CN with 0.1% TFA in 30 min, flow rate 10 mL/min. The fractions containing the desired product were pooled and lyophilized to give pure folded protein **6b** (0.36 mg, 36% yield). The purity and identity of **6b** was confirmed using analytical HPLC and ESI-HRMS. The m/z calculated for **6b** $C_{346}H_{500}N_{81}O_{102}S_8$ $[M+5H]^{5+}$: 1535.6839 Da; measured 1535.6811 Da.

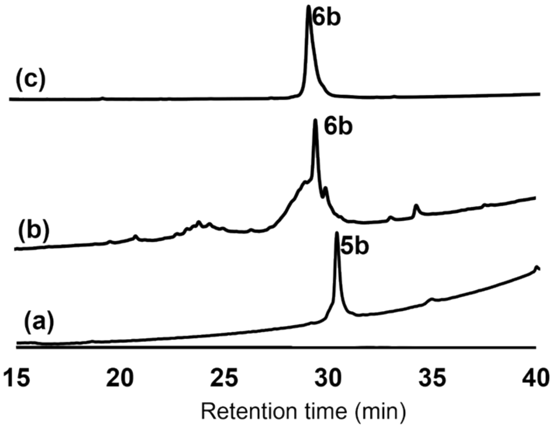

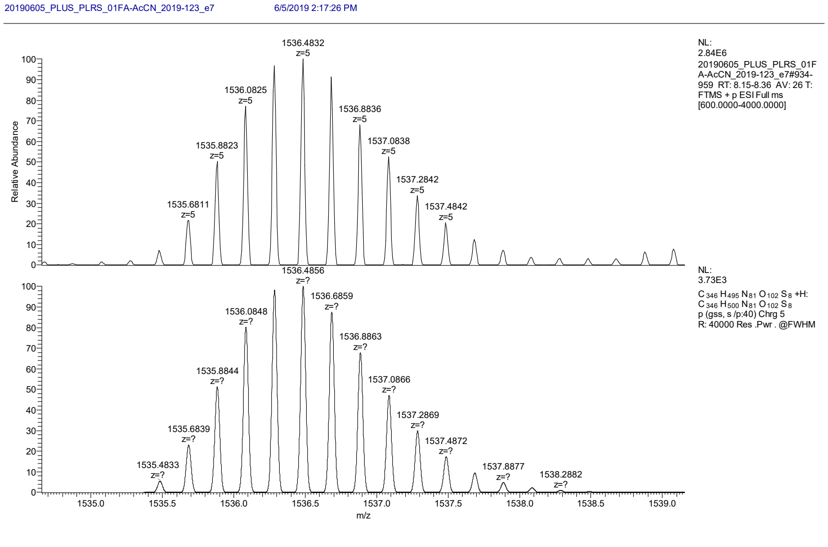

(a) reaction time t = 0 h; (b) reaction time t = 24 h; (c) purified **6b**.

5. Chemical Synthesis of TcLURE Protein by KAHA Ligation

5.1. Synthesis of Cys(Acm) Protected α-Ketoacid Peptide 1b

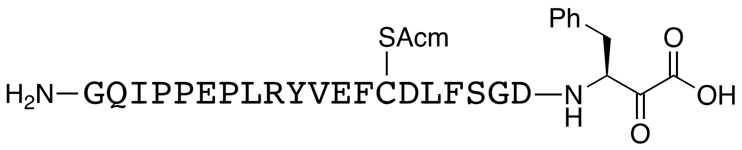

1b

Peptide **1b** was synthesized according to General Procedure 2.1. The crude peptide was purified by preparative HPLC using a Shiseido Capcell pak C18 UG80 (50 mm I.D. × 250 mm) at room temperature with a gradient of 15–65% CH_3CN (with 0.1% TFA) in 20 min, flow rate 40 mL/min and re-purified using a Phenomenex Jupiter C4 (5 µm, 300 Å pore size, 21.2 mm I.D. × 250 mm) at room temperature with a gradient of 20–60% CH_3CN (with 0.1% TFA) in 20 min, flow rate 10 mL/min. The fractions containing the desired product were pooled and lyophilized to give 71 mg of peptide **1b** (obtained from 1 g of dried resin after SPPS). The purity and mass of the peptide was confirmed using analytical HPLC and MALDI respectively. m/z calculated for **1b** $C_{117}H_{168}N_{26}O_{35}S$ $[M+2H]^{2+}$: 1264.5938 Da; measured 1264.5902 Da.

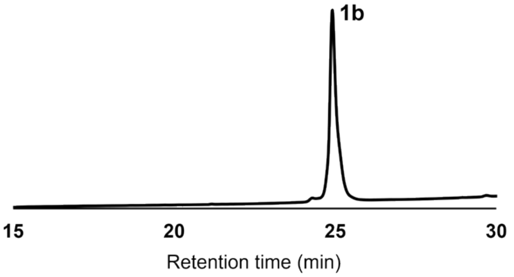

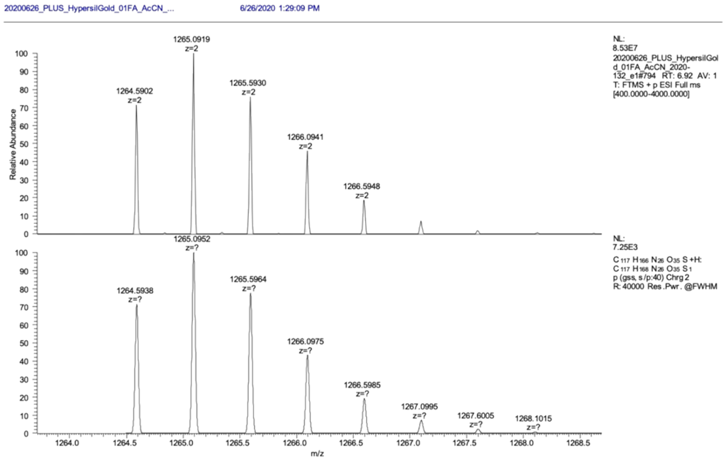

5.2. Synthesis of Cys(Acm) Protected 5-Oxaproline Peptide 2b

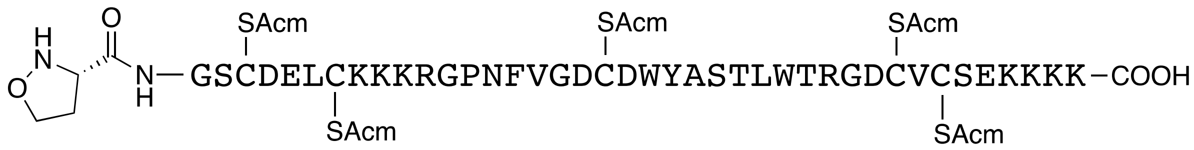

2b

Peptide **2b** was synthesized according to General Procedure 2.2. The crude peptide was purified by preparative HPLC using a Shiseido Capcell pak C18 UG80 (50 mm I.D. × 250 mm) at room temperature with 10–65% CH_3CN (with 0.1% TFA) in 20 min, flow rate 40 mL/min and re-purified by a Phenomenex Jupiter C18 (5 µm, 300 Å pore size, 21.2 mm I.D. × 250 mm) at room temperature with 10–65% CH_3CN (with 0.1% TFA) in 20 min, flow rate 10 mL/min. The fractions containing the desired product were pooled and lyophilized to give 106 mg of desired peptide **2b** (obtained from 1 g of dried resin after SPPS). The purity and identity of **2b** was confirmed using analytical HPLC and ESI-HRMS. The m/z calculated for **2b** $C_{213}H_{343}N_{62}O_{67}S_5$ $[M+7H]^{7+}$:714.4843 Da; measured 714.4816 Da.

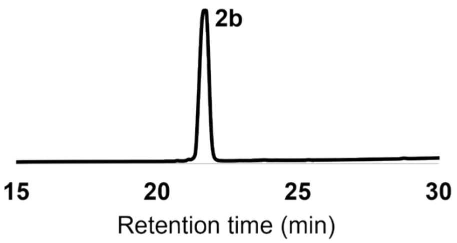

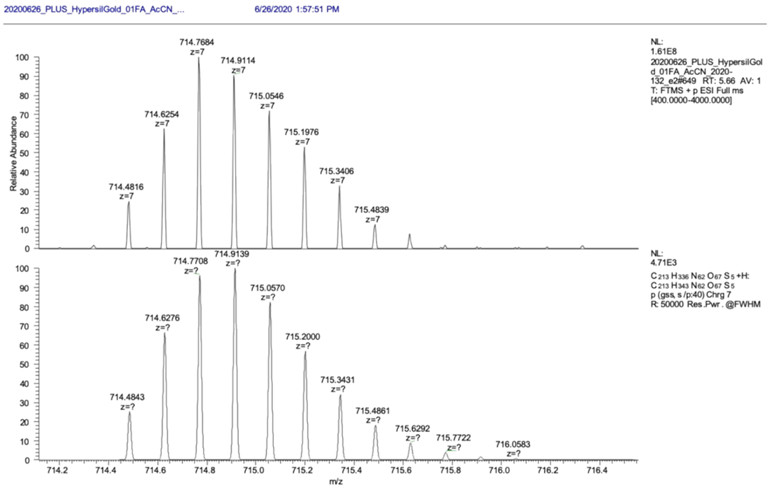

5.3. Synthesis of Cys(Acm) Protected Linear Protein 4c by KAHA Ligation

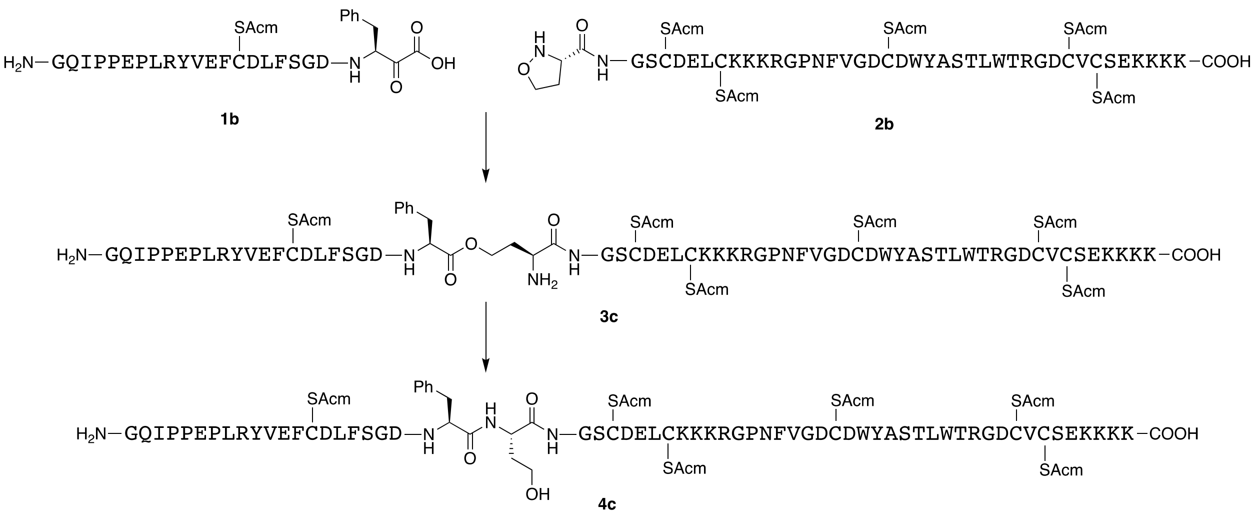

Linear protein **4c** was synthesized according to General Procedure 2.4 using peptide **2b** (20 mg, 5 μmol, 1.0 equiv) and peptide **1b** (30 mg, 6.0 μmol, 1.2 equiv). The reaction mixture was purified by preparative RP-HPLC on a Phenomenex Jupiter C4 (5 μm, 300 Å pore size, 21.2 mm I.D. × 250 mm), heated to 60 °C, with a gradient of 10–60% CH_3CN with 0.1% TFA in 30 min, flow rate 10 mL/min. The fractions containing the desired product were pooled and lyophilized to give pure Acm protected protein **4c** (33 mg, 72% yield). The purity and identity of **4c** was confirmed using analytical HPLC and ESI-HRMS. The m/z calculated for **4c** $C_{329}H_{510}N_{88}O_{100}S_6$ $[M+8H]^{8+}$:935.8230 Da; measured 935.8219 Da.

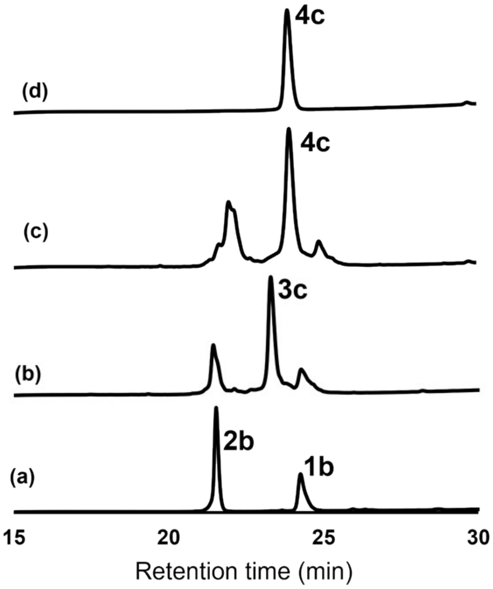

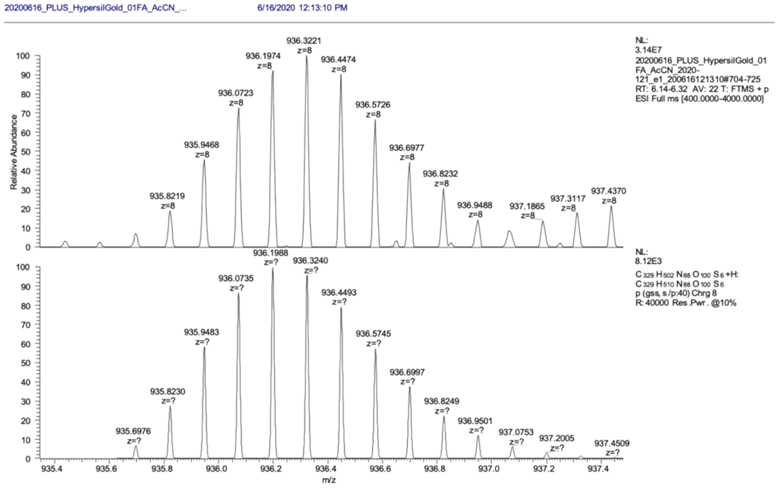

(a) reaction time t = 0 h; (b) reaction time t = 24 h; (c) 2 h after addition of 6M Gdn.HCl solution pH 9.6; (d) purified **4c**.

5.4. Synthesis of Reduced Linear Protein 5c

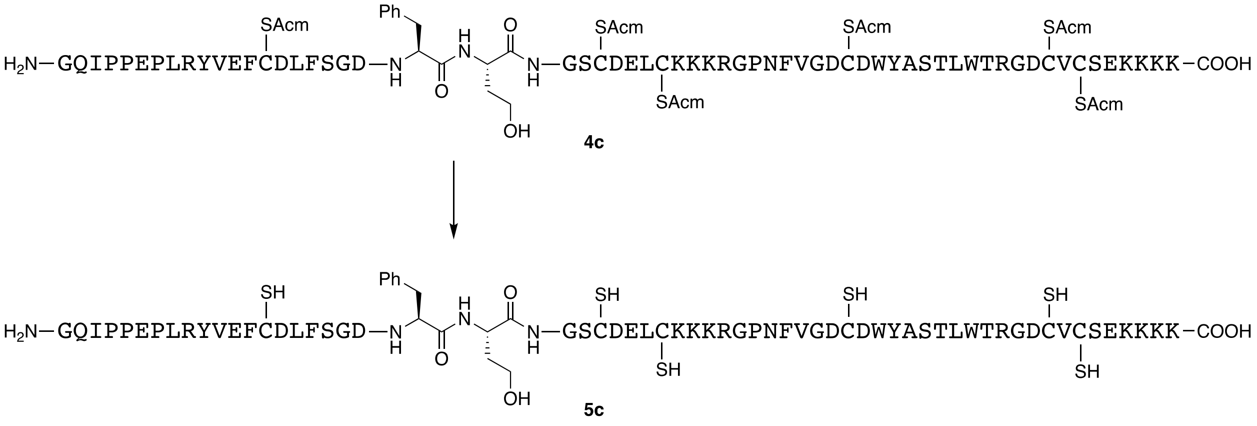

Reduced linear protein **5c** was synthesized according to General Procedure 2.5 with Cys(Acm) protected protein **4c** (10 mg, 1.3 µmol, 1.0 equiv). The obtained mixture was purified by preparative HPLC using a Phenomenex Jupiter C18 (5 µm, 300 Å pore size, 21.2 mm I.D. × 250 mm) at room temperature with a gradient of 30–80% CH_3CN with 0.1% TFA in 30 min, flow rate 10 mL/min. The fractions containing the desired product were pooled and lyophilized to give pure **5c** (6.1 mg, 65% yield). The purity and identity of **5c** was confirmed using analytical HPLC and ESI-HRMS. The m/z calculated for **5c** $C_{311}H_{479}N_{82}O_{94}S_6[M+7H]^{7+}$: 1008.3385 Da; measured 1008.3353 Da.

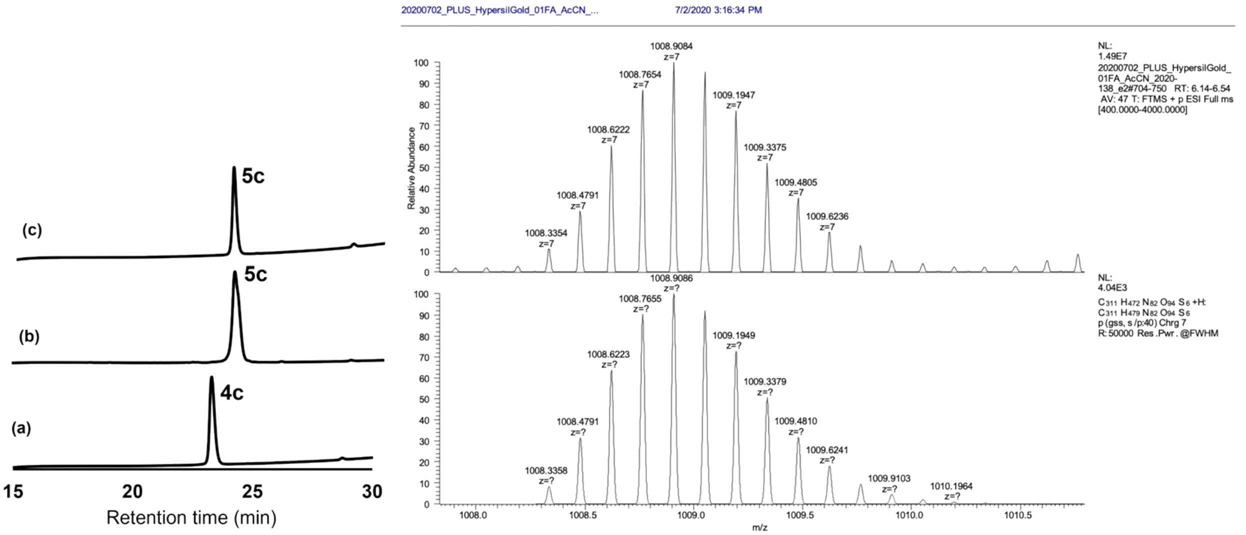

(a) reaction time t = 0 h; (b) reaction time t = 2 h; (c) purified **5c**.

5.5. Synthesis of Folded TcLURE Protein 6c

Folded TcLURE protein **6c** was synthesized according to General Procedure 2.6 with 1.0 mg of reduced linear protein **5c**. The resulting solution was purified by preparative HPLC using a Phenomenex Jupiter C4 (5 µm, 300 Å pore size, 21.2 mm I.D. × 250 mm) with a gradient of 30–80% CH_3CN with 0.1% TFA in 30 min, flow rate 10 mL/min. The fractions containing the desired product were pooled and lyophilized to give pure folded TcLURE **6c** (0.24 mg, 24% yield). The purity and identity of **6c** was confirmed using analytical HPLC and ESI-HRMS. The m/z calculated for **6c** $C_{311}H_{474}N_{82}O_{94}S_6$ $[M+8H]^{8+}$: 881.7892 Da; measured 881.7881 Da.

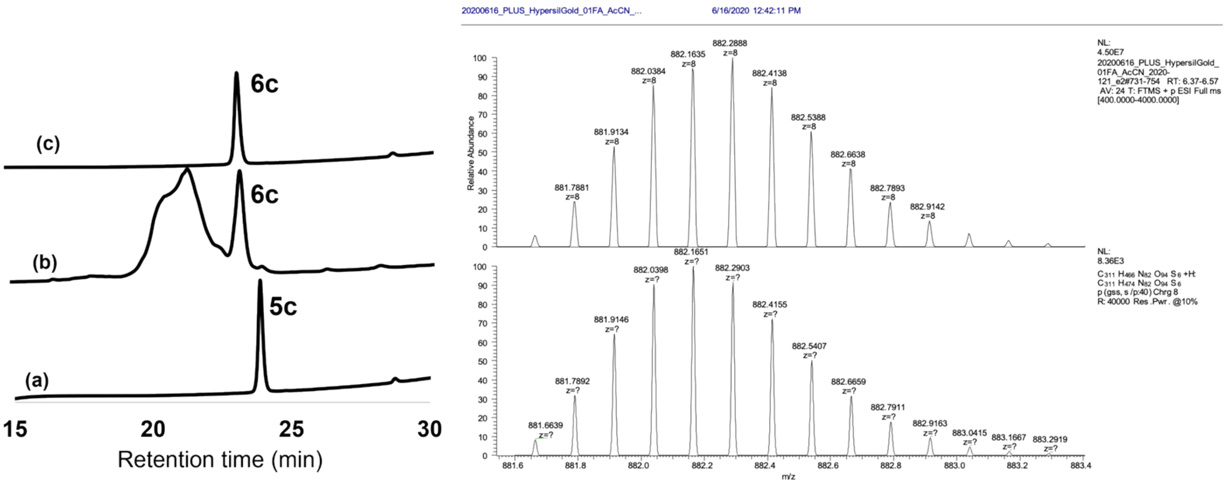

(

a) reaction time t = 0 h; (b) reaction time t = 24 h; (c) purified **6c**.

Folded TcLURE protein 6c was dissolved in 25 mM of potassium phosphate buffer (pH = 7.0) and the CD spectrum was measured by a J-1500 spectropolarimeter (JASCO, Tokyo) at room temperature using 0.1 cm cell.

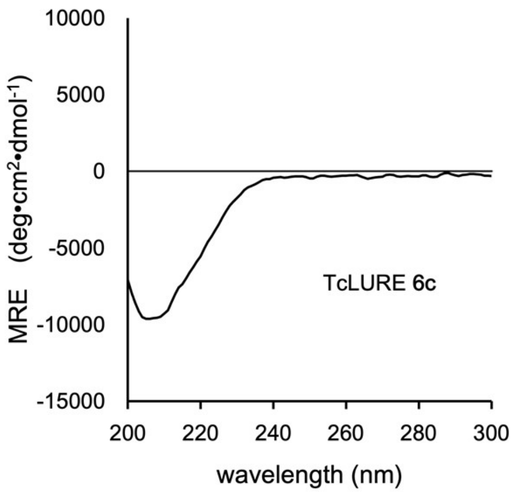

6. Chemical Synthesis of Analogues of LURE Proteins by KAHA Ligation

6.1. Synthesis of Cys(Acm) Protected Linear Protein 4d by KAHA ligation

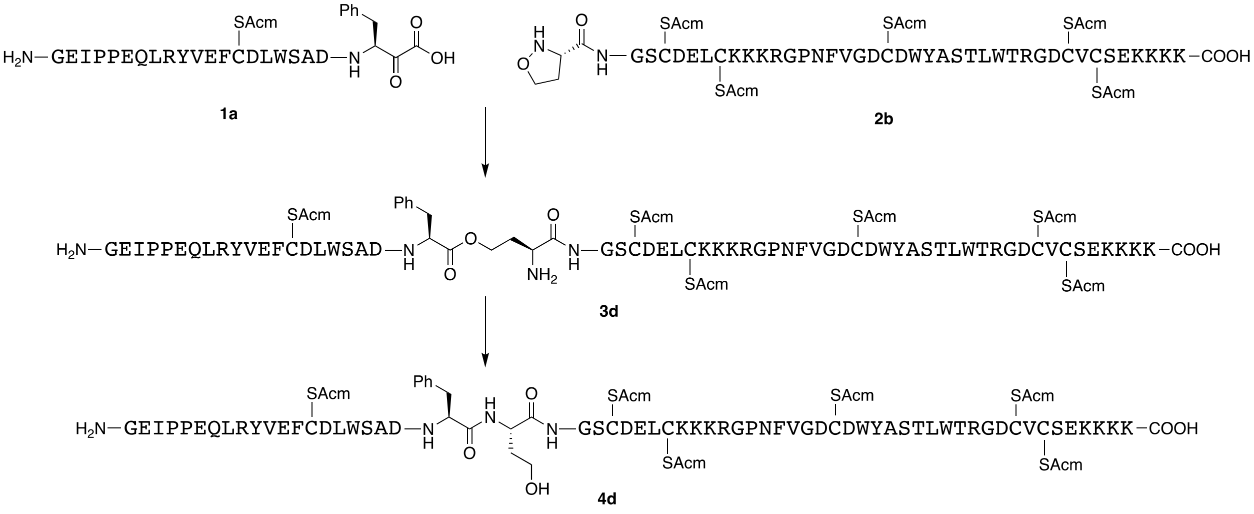

Linear protein **4d** was synthesized according to General Procedure 2.4 using peptide **2b** (30 mg, 6.0 μmol, 1.0 equiv) and peptide **1a** (9.0 mg, 7.2 μmol, 1.2 equiv). The reaction mixture was purified by preparative RP-HPLC on a Phenomenex Jupiter C18 (5 μm, 300 Å pore size, 21.2 mm I.D. × 250 mm), at room temperature; with a gradient of 10–60% CH_3CN with 0.1% TFA in 30 min, flow rate 10mL/min. The fractions containing the desired product were pooled and lyophilized to give pure Acm protected protein **4d** (31 mg, 67% yield over two steps). The purity and identity of **4d** was confirmed using analytical HPLC and ESI-HRMS. The m/z calculated for **4d** $C_{332}H_{513}N_{89}O_{102}S_6$ $[M+8H]^{8+}$:946.4500 Da; measured 946.4469 Da.

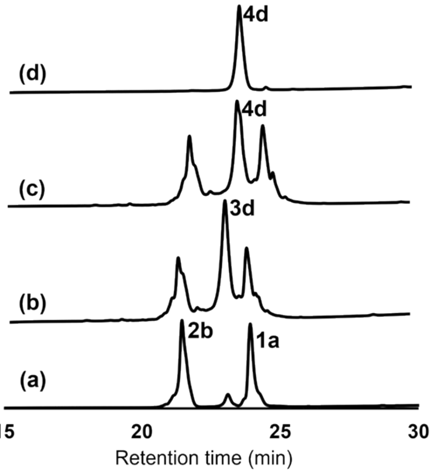

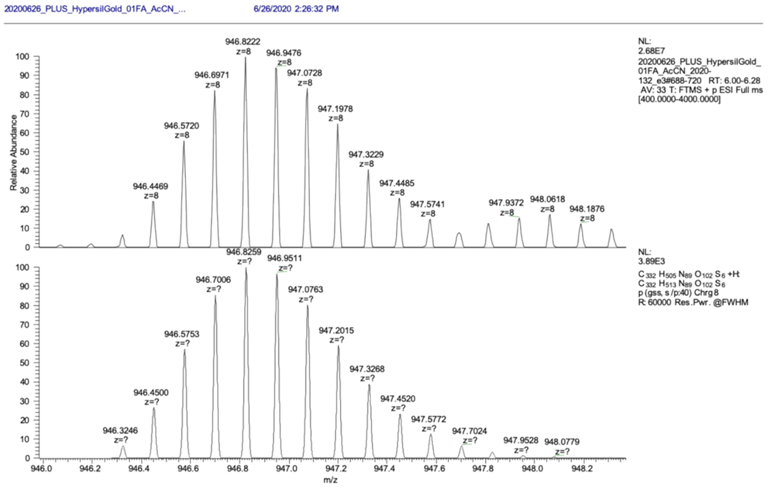

(a) reaction time t = 0 h; (b) reaction time t = 24 h; (c) 2 h after addition of 6M Gdn.HCl solution pH 9.6; (d) purified **4d**.

6.2. Synthesis of Linear Protein 5d

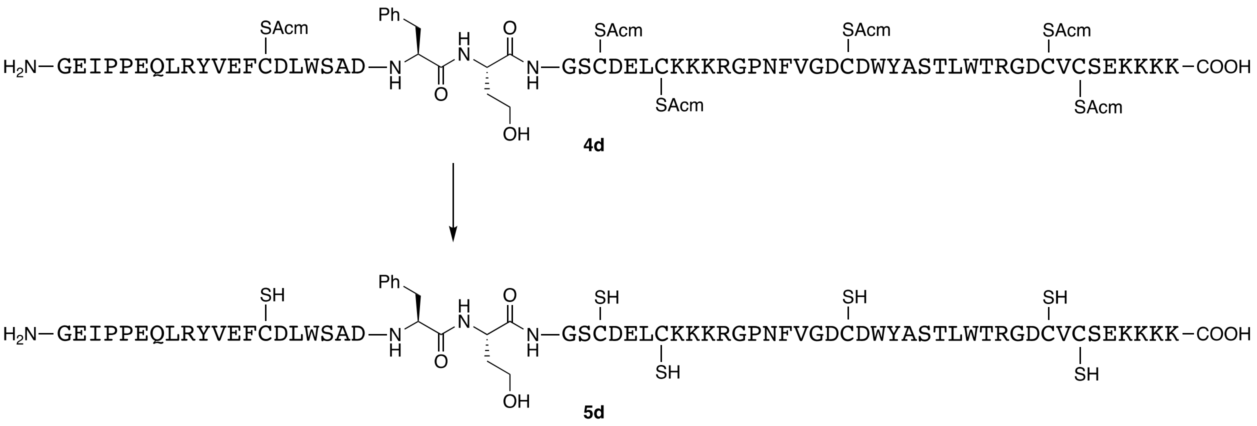

Reduced linear protein **5d** was synthesized according to General Procedure 2.5 with Cys(Acm) protected protein **4d** (10 mg, 1.32 μmol, 1.0 equiv). The obtained mixture was purified by preparative HPLC using a on a Phenomenex Jupiter C4 (5 μm, 300 Å pore size, 21.2 mm I.D. × 250 mm) at 25 °C, with a gradient of 30–80% CH_3CN with 0.1% TFA in 30 min, flow rate 10 mL/min. The fractions containing the desired product were pooled and lyophilized to give pure **5d** (6.4 mg, 68% yield). The purity and identity of **5d** was confirmed using analytical HPLC and ESI-HRMS. The m/z calculated for **5d** $C_{314}H_{482}N_{83}O_{96}S_6[M+7H]^{7+}$: 1020.6243 Da; measured 1020.6245 Da.

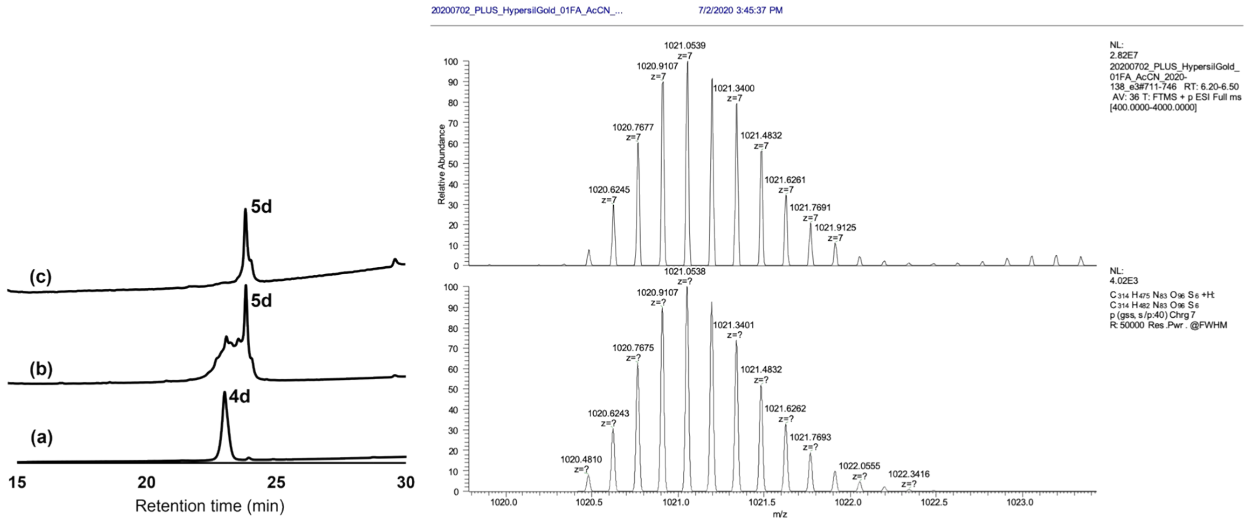

(a) reaction time t = 0 h; (b) reaction time t = 2 h; (c) purified **5d**.

6.3. Synthesis of Folded TfTcLURE Protein 6d

Folded TfTcLURE protein **6d** was synthesized according to General Procedure 2.6 with 1.0 mg of reduced linear protein **5d**. The resulting solution was purified by preparative HPLC using a Phenomenex Jupiter C4 (5 µm, 300 Å pore size, 21.2 mm I.D. × 250 mm) with a gradient of 20–80% CH_3CN with 0.1% TFA in 30 min, flow rate 10 mL/min. The fractions containing the desired product were pooled and lyophilized to give pure folded TfTcLURE **6d** (0.27 mg, 27% yield). The purity and identity of **6d** was confirmed using analytical HPLC and ESI-HRMS. m/z calculated for **6d** $C_{314}H_{477}N_{83}O_{96}S_6$ $[M+8H]^{8+}$: 892.4163 Da; measured 892.4154 Da.

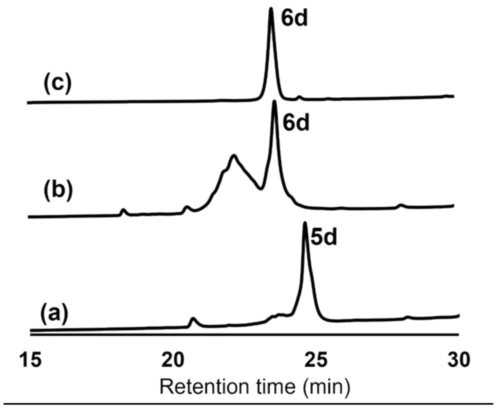

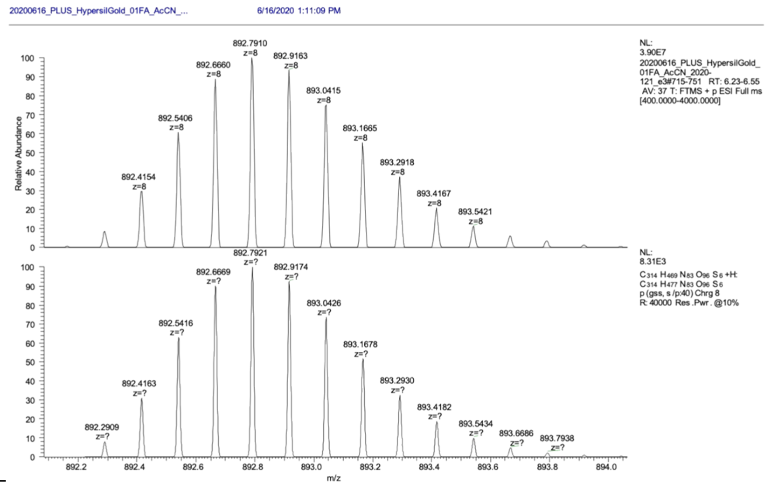

(a) reaction time t = 0 h; (b) reaction time t = 24 h; (c) purified **6d**.

Folded TfTcLURE protein 6d was dissolved in 25 mM of potassium phosphate buffer (pH = 7.0) and the CD spectrum was measured by a J-1500 spectropolarimeter (JASCO, Tokyo) at room temperature using 0.1 cm cell.

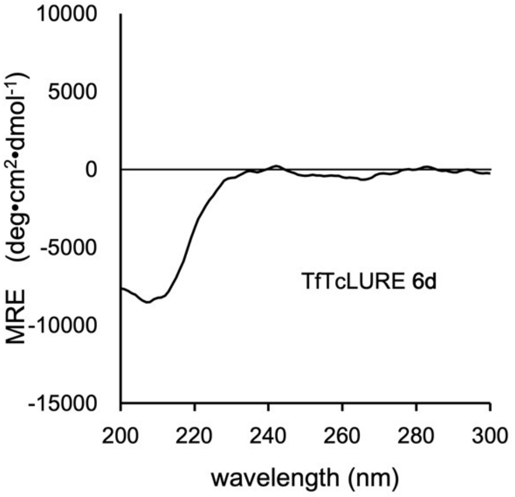

6.4. Synthesis of Cys(Acm) Protected Linear Protein 4e by KAHA ligation

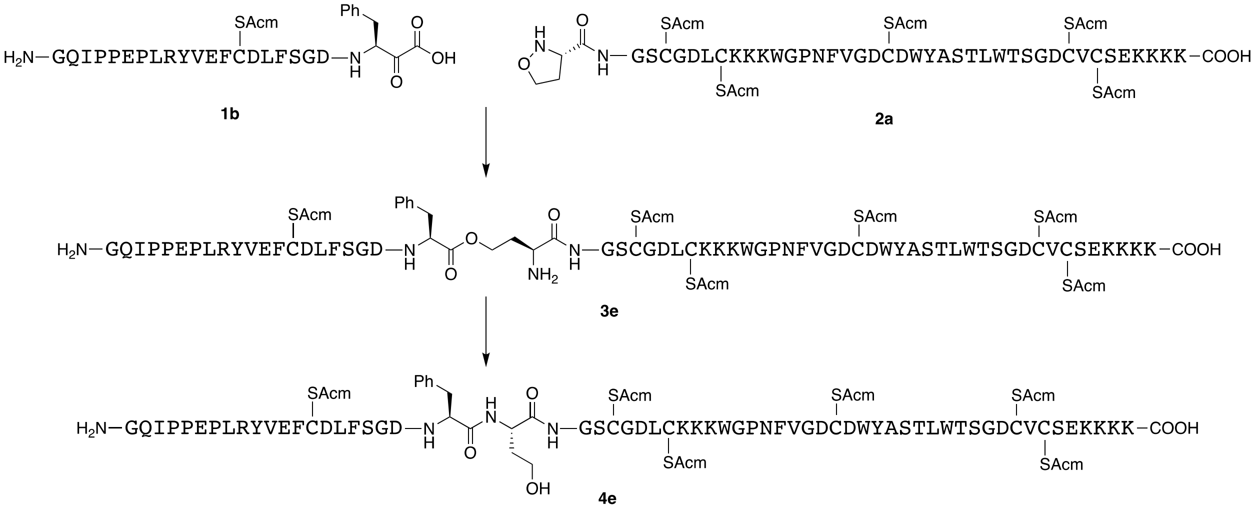

Linear protein **4e** was synthesized according to General Procedure 2.4 using peptide **2a** (30 mg, 6.2 μmol, 1.0 equiv) and peptide **1b** (19 mg, 7.4 μmol, 1.2 equiv). The reaction mixture was purified by preparative RP-HPLC on a Phenomenex Jupiter C4 (5 μm, 300 Å pore size, 30 mm I.D. × 250 mm) at room temperature with a gradient of 10–70% CH_3CN with 0.1% TFA in 30 min, flow rate 20 mL/min. The fractions containing the desired product were pooled and lyophilized to give pure Acm protected protein **4e** (28 mg, 60% yield over two steps). The purity and identity of **4e** was confirmed using analytical HPLC and ESI-HRMS. The m/z calculated for **4e** $C_{328}H_{497}N_{83}O_{99}S_6$ $[M+8H]^{8+}$: 921.9340 Da; measured 921.9304 Da.

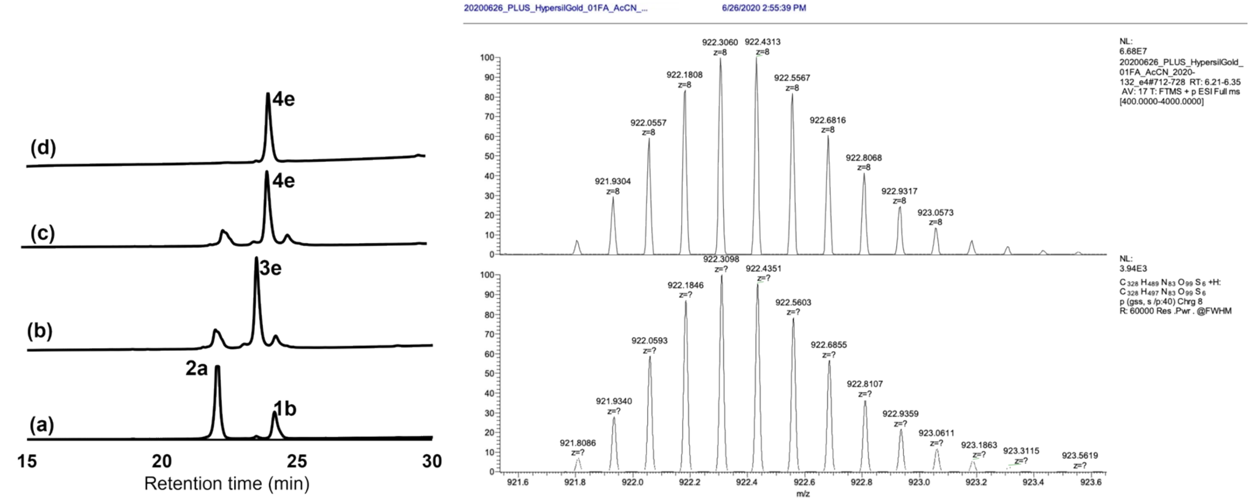

(a) reaction time t = 0 h; (b) reaction time t = 24 h; (c) 2 h after addition of 6M Gdn•HCl solution pH 9.6; (d) purified **4e**.

6.5. Synthesis of Linear Protein 5e

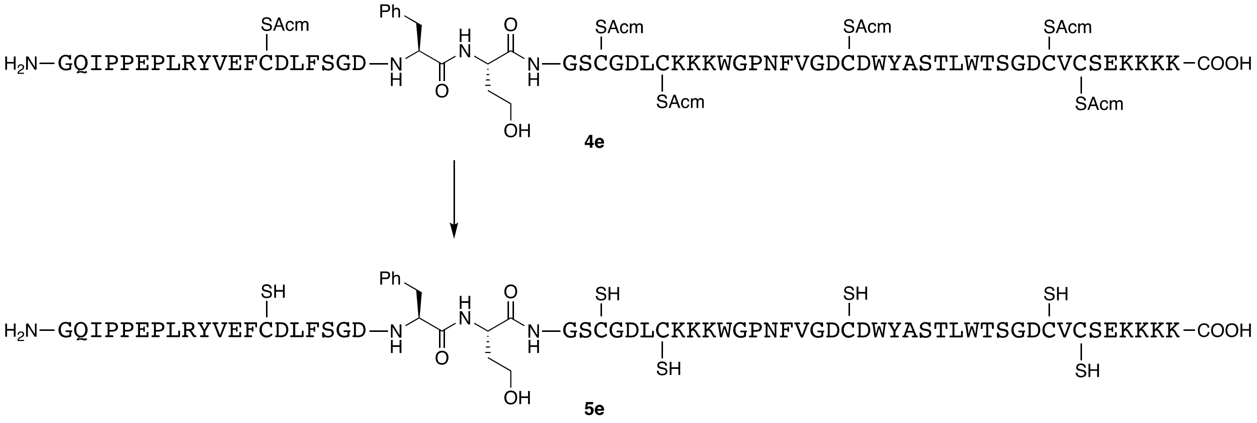

Reduced linear protein **5e** was synthesized according to General Procedure 2.5 with Cys(Acm) protected protein **4e** (10 mg, 1.35 μmol, 1.0 equiv). The obtained mixture was purified by preparative HPLC using a Phenomenex Jupiter C18 (5 μm, 300 Å pore size, 21.2 mm I.D. × 250 mm) at room temperature, with a gradient of 30–80% CH_3CN with 0.1% TFA in 30 min, flow rate 10 mL/min. The fractions containing the desired product was pooled and lyophilized to give pure **5e** (6.8 mg, 72% yield). The purity and identity of **5e** was confirmed using analytical HPLC and ESI-HRMS. The m/z calculated for **5e** $C_{310}H_{466}N_{77}O_{93}S_6$ $[M+7H]^{7+}$: 992.6060 Da; measured 992.6057 Da.

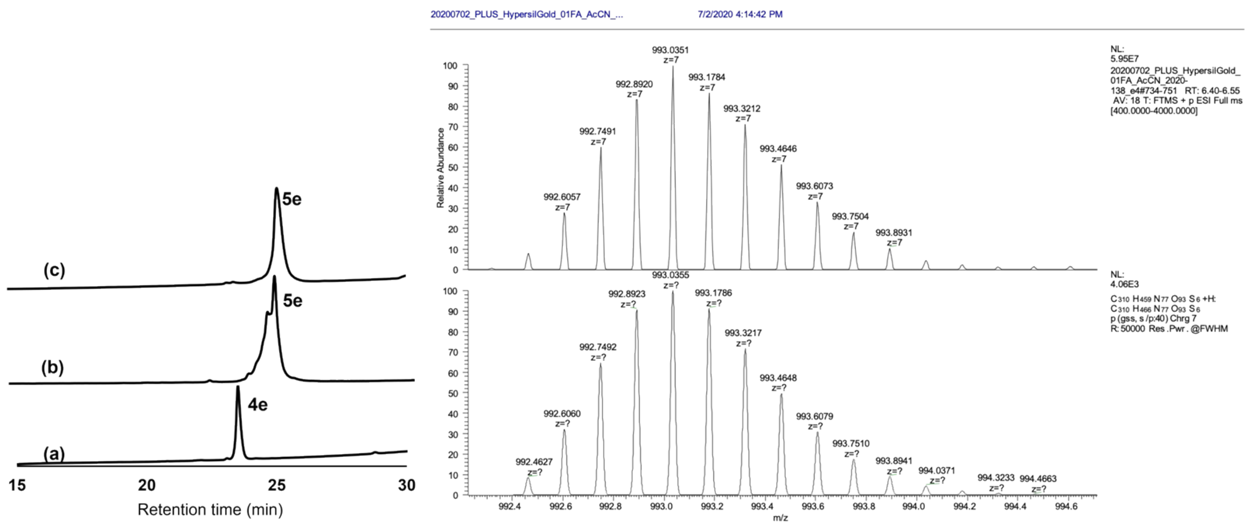

(a) reaction time t = 0 h; (b) reaction time t = 2 h; (c) purified **5e**.

6.6 Synthesis of Folded TcTfLURE Protein 6e

Folded TcTfLURE protein **6e** was synthesized according to General Procedure 2.6 with 1.0 mg of reduced linear protein **5e**. The resulting solution was purified by preparative HPLC using a on a Phenomenex Jupiter C18 (5 µm, 300 Å pore size, 21.2 mm I.D. × 250 mm) with a gradient of 10–70% CH_3CN with 0.1% TFA in 30 min, flow rate 10 mL/min. The fractions containing the desired product was pooled and lyophilized to give pure folded TcTfLURE **6e** (0.30 mg, 30% yield). The purity and identity of **6e** was confirmed using analytical HPLC and ESI-HRMS. m/z calculated for **6e** $C_{310}H_{454}N_{77}O_{93}S_6$ $[M+7H]^{7+}$: 991.7421 Da; measured 991.7405 Da.

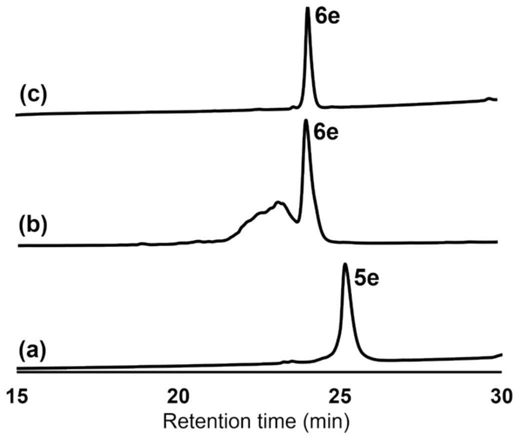

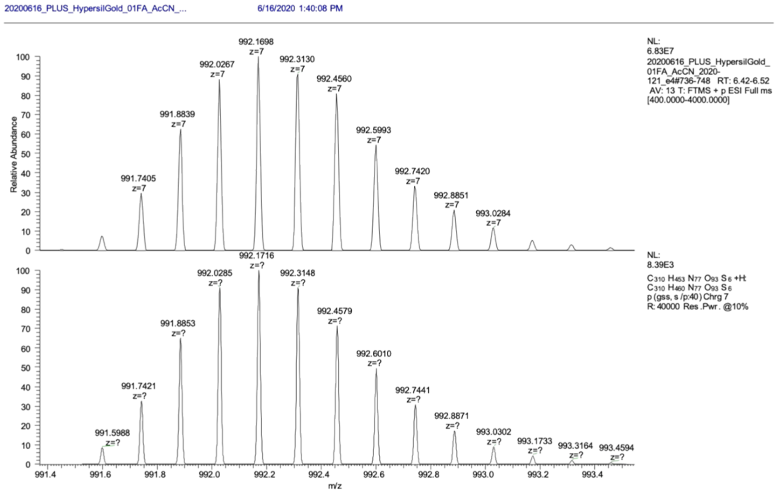

(a) reaction time t = 0 h; (b) reaction time t = 24 h; (c) purified **6e**

Folded TcTfLURE protein 6e was dissolved in 25 mM of potassium phosphate buffer (pH = 7.0) and the CD spectrum was measured by a J-1500 spectropolarimeter (JASCO, Tokyo) at room temperature using 0.1 cm cell.

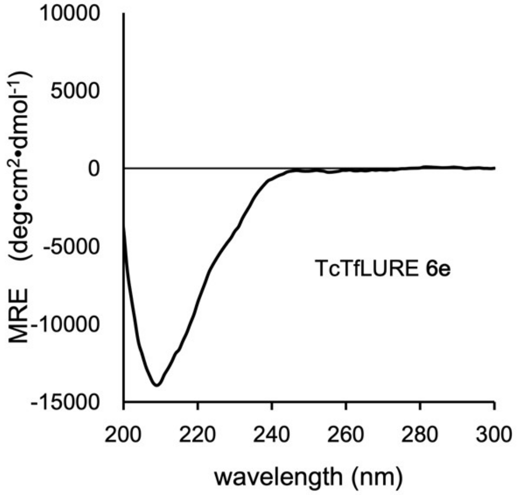

7. Analysis of LURE Proteins by SDS-Page

analyzed by SDS-PAGE (100 V, BIO-RAD mini-PROTEAN Tris-Tricine precast gel).

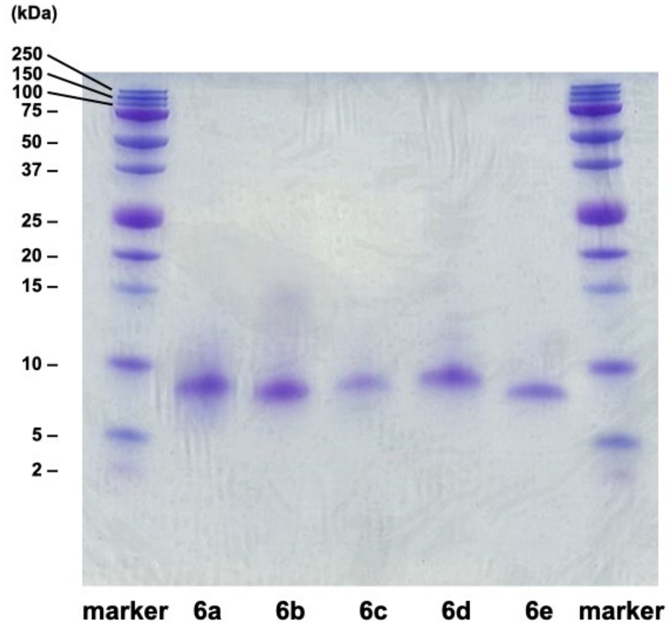

8. References

1 T. G. Wucherpfennig, V. R. Pattabiraman, F. R. P. Limberg, J. Ruiz-Rodríguez and J. W. Bode, *Angew. Chem., Int. Ed.*, **2014**, *53*, 12248–12252.

2 C. E. Murar, T. J. Harmand, H. Takano, J. W. Bode, *Org. Synth*. **2018**, *95*, 157–176
